# Polyglycerol‐Based Lipids: A Next‐Generation Alternative to PEG in Lipid Nanoparticles for Advanced Drug Delivery Systems

**DOI:** 10.1002/marc.202500428

**Published:** 2025-09-16

**Authors:** Yara Ensminger, Rashmi Rashmi, Michael Karimov, Gideon Nölte, Markus Hafke, Ann‐Cathrin Schmitt, David Díaz‐Oviedo, Johannes Köbberling, Rainer Haag

**Affiliations:** ^1^ Institute For Chemistry and Biochemistry Freie Universität Berlin Berlin Germany; ^2^ Chemical and Pharmaceutical Development Bayer AG Pharmaceuticals Berlin Germany; ^3^ Drug Discovery Sciences Bayer AG Pharmaceuticals Wuppertal Germany

**Keywords:** gene delivery, lipid nanoparticles, peg‐replacement, polyglycerol

## Abstract

Polyethylene glycol (PEG) is widely used to provide a stealth effect in various therapeutic applications. However, an increased occurrence of anti‐PEG antibodies in patients can lead to immunological side effects and accelerated blood clearance, reducing the efficiency of PEG‐based delivery vectors. One of these PEG‐containing vectors is lipid nanoparticles (LNPs), which effectively deliver nucleic acids. Their high potential for treating a great number of diseases in the future was already showcased in the mRNA vaccines developed during the COVID‐19 pandemic. Thus, the aim of our study was to formulate LNP systems utilizing linear polyglycerol (lPG) as an alternative stealth polymer to avoid anti‐PEG antibodies while enabling mRNA delivery. Our study showed that lPG‐functionalized LNPs had negligible binding to IgG anti‐PEG antibodies, while successfully delivering eGFP mRNA into HepG2 cells with comparable transfection efficiency as PEGylated LNPs.

## Graphical abstract

Linear polyglycerol is proposed as a PEG‐alternative in mRNA‐loaded lipid nanoparticles to limit activation of anti‐PEG antibodies and reduce immunogenicity. Resulting nanoparticles may provide similar gene delivery capabilities, while preventing binding of anti‐PEG antibodies compared to PEGylated counterparts.

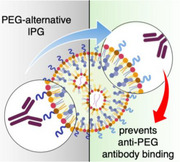

## 1 Introduction

Polyethylene glycol (PEG) has long been a staple in the field of drug delivery due to its ability to improve the pharmacokinetics and biocompatibility of therapeutic agents. The role of PEGylated lipids in the recent coronavirus pandemic was pivotal for the development of mRNA‐based vaccines, a promising therapy approach for a variety of diseases. PEGylated lipids are a key component of lipid nanoparticles (LNPs), used to encapsulate and deliver mRNA vaccines, such as the Pfizer‐BioNTech and Moderna COVID‐19 vaccines [1, 2]. These LNPs protect the delicate mRNA molecules, enhance their stability, and facilitate delivery to target cells that ultimately produce target proteins to combat a disease of interest. Commercial LNP formulations frequently contain four different lipids: ionizable lipids, helper lipids, cholesterol, and stabilizer lipids (often PEG‐based), to provide colloidal stability and stealth effects [3, 4, 5]. However, despite their crucial role in mRNA vaccines, PEG‐lipids have several disadvantages, with concerns regarding their immunogenicity, potential toxicity, or non‐biodegradability [6]. Amplifying these concerns, a drastic increase in anti‐PEG antibody concentrations in recent decades has been linked to allergic reactions, anaphylactic shock, and may even reduce vaccine efficiency with repeated administration due to pre‐existing immunity to PEG [7, 8, 9, 10, 11, 12, 13]. These anti‐PEG antibodies may have a higher affinity for the polymer backbone, or may target the methoxy end group of mPEG, a PEG‐variation frequently used for therapeutic formulations [10, 14]. Furthermore, Lee et al., 2024 recently found that PEG attached to LNPs induced a stronger IgM anti‐PEG antibody response than free PEG alone [11]. Addressing these challenges is essential for optimizing vaccine safety, efficacy, and accessibility to establish mRNA‐based therapies for a broad variety of diseases.

In recent years, a growing body of research has focused on exploring PEG‐alternatives in advanced drug delivery systems, including LNPs. Such alternatives span polysarcosines (pSar), poly(2‐oxazoline)s (pOx), and linear polyglycerol (lPG). As a structural derivative of glycerol, lPG is a biocompatible and highly functionalizable polymer, and was therefore the focus of our study. Like PEG, lPG is a hydrophilic polymer, composed of multiple glycerol units linked by ether bonds [13, 15, 16]. Its unique structure offers several advantages over PEG, including enhanced stability, reduced immunogenicity, and improved biodegradability. Additionally, specific properties—such as size, molecular weight, and surface functionality—can be tailored, making it highly versatile for various drug delivery applications [17, 18, 19, 20]. Such polymers have garnered significant interest due to their ability to encapsulate a wide range of therapeutic agents, including small molecules, peptides, proteins, and nucleic acids, enabling delivery to target tissues with improved efficiency and reduced side effects. Our research focuses on establishing lPG as a PEG alternative in the formulation of mRNA‐encapsulating LNPs, with a focus on varying lPG chain lengths and methoxy‐functionalized lPG. These structurally similar polymers offer insights into how hydrophilic and amphiphilic polymers may influence lipid structure within LNPs.

A main constituent of commercial mRNA‐LNP formulations is DMG‐PEG2000 (as used in Moderna's SpikeVax [2]), providing a stealth effect and limiting immunogenicity upon administration. These polymer‐lipids consist of a mPEG chain (average MW 2000 g mol^−1^) featuring a terminal methoxy group and are covalently linked to dimyristoyl glycerol via an ether linkage. While effective, the resulting mPEG‐lipids are susceptible to detection and binding by anti‐PEG antibodies, potentially causing antigenic responses in PEG‐sensitive patients [21, 22]. The increasing prevalence of these antibodies and ensuing effects motivated us to investigate lPG‐derivatives as PEG‐alternatives for mRNA‐loaded LNP vaccines. To address this, we grafted different lPG structures to myristoyl glycerol via an ether linkage, enabling direct comparison with DMG‐PEG2000 when incorporated into LNPs.

The lPG variations were designed to replicate either the molecular weight or the chain length of PEG2000, and all featured at least one terminal methoxy group. The resulting lPG with comparable chain length and no additional methoxy groups may be deemed structurally most similar to PEG. Additionally, MeOlPG, lPG‐*block*‐MeOlPG, and MeOlPG‐*block*‐lPG were modified to include methoxy groups along the polymer backbone. MeOlPG was employed to assess LNPs utilizing a simpler polymer synthesis. By varying the order of functional groups in lPG‐*block‐*MeOlPG and MeOlPG‐*block*‐lPG, we hoped to gain better insights into additional polarity and potential steric hindrance along the polymer backbone. Each lPG variant was covalently linked to two C‐14 alkyl chains, mirroring the lipid tails of DMG and providing an amphiphilic character. Resulting amphiphilic polymers were then incorporated into mRNA‐LNP formulations to assess the influence of polymer properties (such as polarity and chain length) on LNP structures. Finally, the delivery efficiency of these lPG‐containing LNPs was quantified by encapsulating eGFP mRNA, to produce eGFP after intracellular release and mRNA translation.

## 2 Results and Discussion

This study aimed to investigate lPG as an alternative to PEG in mRNA LNP systems, to limit binding of anti‐PEG antibodies while maintaining stealth effects and transfection efficiency. To this extent, we synthesized different lPG‐derivative amphiphiles with varying polarity and chain length. These amphiphiles were subsequently formulated into mRNA LNP systems, which were then analyzed in terms of mRNA delivery and intracellular transfection.

### 2.1 Synthesis of Polyglycerol Lipids

**FIGURE 1:**
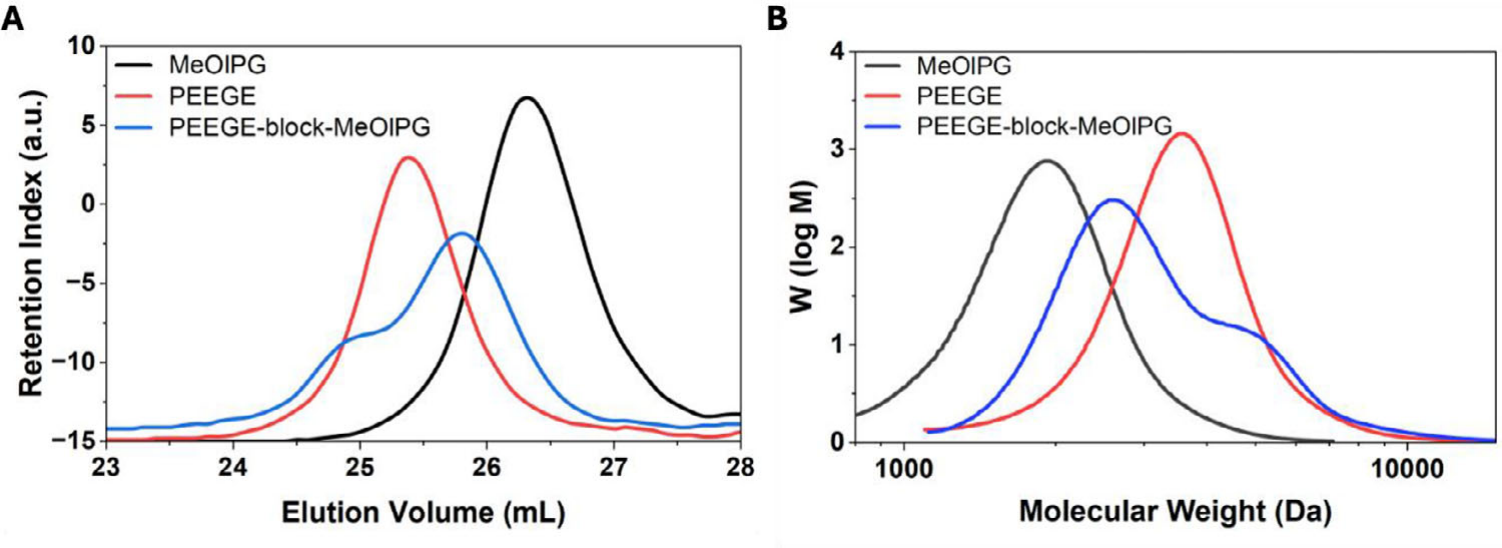
GPC analysis of homopolymers and block copolymer, showing (a) Molecular weight distributions of protected homopolymers EEGE (2b) and MeOlPG (3b), as well as block copolymer EEGE‐block‐MeOlPG (4b), plotted as weight fraction versus molecular weight; and (b) corresponding GPC elution curves (RI detector). Samples were measured in THF with PS calibration.

The polymeric backbones for this study (lPG and MeOlPG) were prepared by living anionic polymerization with 2,3‐bis(benzyloxy)propan‐1‐ol as initiator via reaction with the respective oxirane monomer, followed by debenzylation via hydrogenation in toluene under dry conditions. Gel permeation chromatography (GPC) was used to determine molecular weight and the corresponding number of repeating units for each polymer prior to functionalization with double ester moieties (Figure 1). PEEGE showed a lower elution volume, consistent with its higher molecular weight (3676 Da). Although the deprotection of PEEGE later in the synthesis reduces the molecular weight, the number of repeating units remains unchanged and can be inferred from this data. Successful formation of the block copolymers was confirmed by their elution peak appearing between those of the homopolymers (Figure 1b) and their molecular weight distribution spanning both homopolymers (Figure 1a).

**SCHEME 1:**
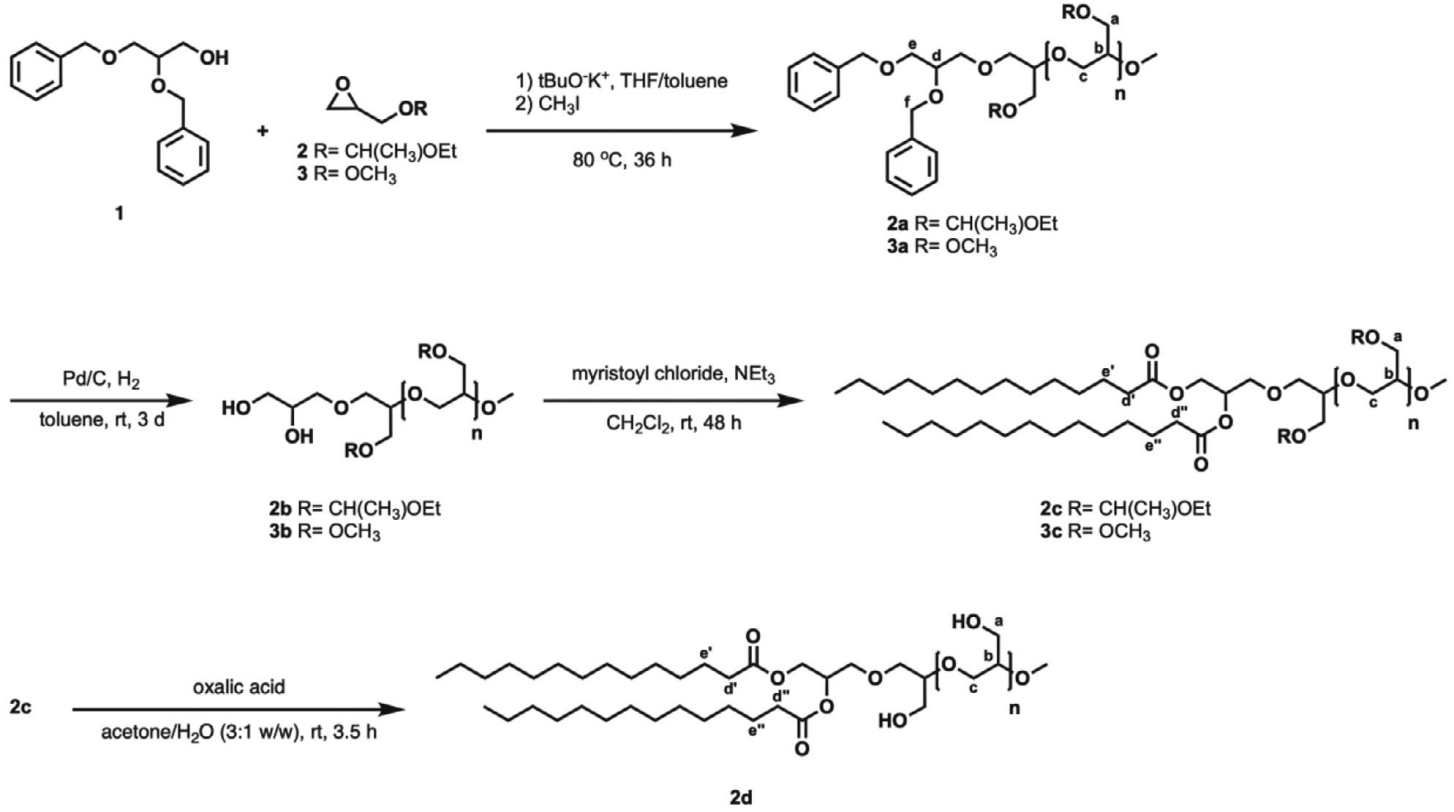
Synthesis of lPG and MeOlPG‐based polymeric lipids.

**SCHEME 2:**
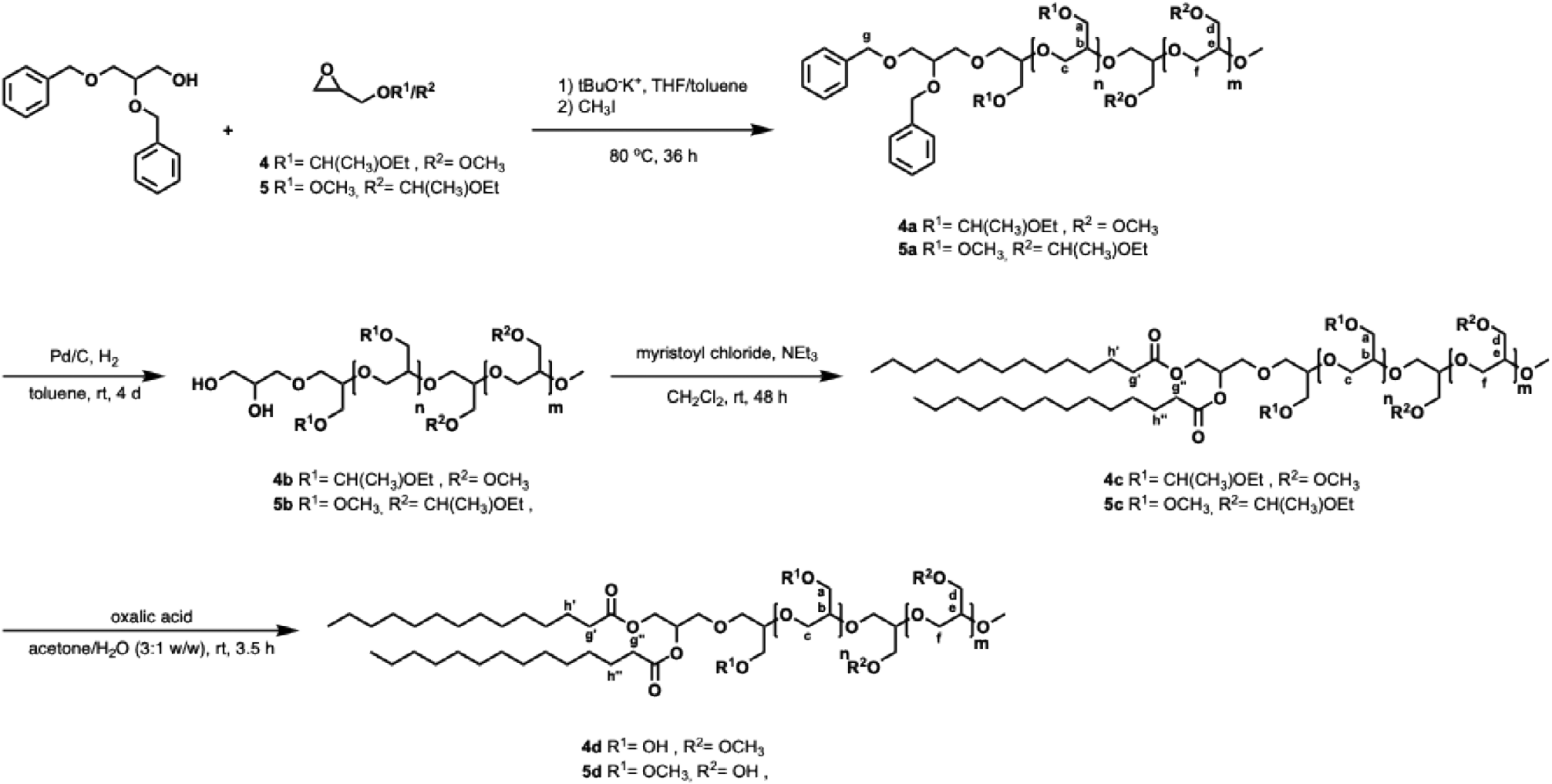
Synthesis of copolymeric lPG‐block‐MeOlPG and MeOlPG‐block‐lPG ‐based polymeric lipids.

After molecular weight determination, the diol‐functionalized lPGs were coupled with myristoyl chloride to obtain the double ester moieties. For the case of EEGE‐containing polymers, the hydroxyl side chains were obtained by deprotection of the acetal‐protected side chains (Scheme 1 and Table 1). The copolymers were prepared by a similar route, only involving a stepwise addition of the different oxirane monomers during the ring‐opening polymerization step (Scheme 2 and Table 1). All synthesized polymers were characterized by spectroscopic techniques.

**TABLE 1:** Overview of molecular weight, chain lengths, and functional groups of PEG and lPG polymers used.

Polymer	MW [Da]	# of repeating units	Functional groups
DMG‐PEG2000	2509	44	—
DMG‐lPG (**2d**)	3119	42	OH
DMG‐MeOlPG (**3c**)	2553	27	OCH_3_
DMG‐lPG‐*b*‐MeOlPG (**4d**)	3060	n_(OH)_ = 18; m_(OCH3)_ = 13	R^1^ = OH; R^2^ = OCH_3_
DMG‐MeOlPG‐*b*‐lPG (**5d**)	3390	n_(OCH3)_ = 19; m_(OH)_ = 22	R^1^ = OCH_3_; R^2^ = OH

### 2.2 Biophysical Characterization of LNPs

#### 2.2.1 Size and Stability of LNPs

The LNPs were formulated by mixing aqueous and organic phases via microfluidics. Negatively charged mRNA in the aqueous phase formed a stabilizing complex with ionizable cationic lipids in the organic phase. Remaining lipids in the organic phase self‐assembled around these complexes to form mRNA‐LNP systems. Finally, ultrafiltration centrifugation was used for buffer replacement to storage buffer, before measuring the size and PDI of the resulting LNP systems using DLS. Desirable nanoparticle diameters were in the 60–150 nm range, to facilitate cellular uptake and produce robust immune responses [23, 24]. Furthermore, a low PDI was desired as an indicator for a homogeneous population with a narrow size distribution. Size of nanoparticles may be decreased by the presence of a stealth lipid, resulting in steric hindrance during self‐assembly and reducing particle agglomeration.

In this study, microfluidic process flow conditions were optimized to render PEG‐LNPs with a mean particle size of 110.0 nm (PDI 0.045) (Table 2). With a chain length akin to PEG2000 (42 vs. 44 repeating units, respectively; Table 1), lPG resulted in the smallest LNPs of all tested lPG‐variations, followed by MeOlPG‐*block*‐lPG. Contrasting, MeOlPG and lPG‐*block*‐MeOlPG's shorter chain lengths provided limited stealth effects, forming significantly larger particles [25]. The differing chain length limits conclusions that can be drawn from comparing the two block copolymers, but may indicate that LNP size is independent of additional functional groups along the lPG backbone.

**TABLE 2:** Overview of particle size, polydispersity index, zeta potential, and encapsulation efficiency of mRNA using PEG and lPG polymers to formulate LNPs.

Polymer	Diameter (nm)	PDI	Zeta potential (mV)	Encapsulation efficiency (%)
DMG‐PEG2000	110.0 ± 6.3	0.045 ± 0.030	−3.30 ± 0.55	98.3 ± 7.5
DMG‐lPG (**2d**)	223.1 ± 24.4	0.105 ± 0.072	−6.05 ± 2.82	93.4 ± 8.5
DMG‐MeOlPG (**3c**)	252.3 ± 14.0	0.169 ± 0.063	−6.78 ± 2.37	61.9 ± 33.2
DMG‐lPG‐*b*‐MeOlPG (**4d**)	264.3 ± 66.5	0.154 ± 0.138	−4.11 ± 2.33	79.4 ± 17.0
DMG‐MeOlPG‐*b*‐lPG (**5d**)	233.7 ± 28.2	0.113 ± 0.027	−4.10 ± 2.13	78.1 ± 23.3

The comparatively larger size of lPG‐ versus PEG‐LNP systems has previously been observed with other PEG‐alternatives (such as pSar‐LNP systems) and is therefore not indicative of a limitation specific to our formulation [26, 27, 28]. Nonetheless, adjustments of production parameters may help reduce lPG‐LNP diameters. By varying microfluidic settings (e.g., microfluidic chip conformation, flow rate/ratio) or types of aqueous buffer, previous works have shown that a reduction in nanoparticle size is possible [4, 29]. Increasing molar amounts of stealth lipid can decrease LNP size but comes at the expense of reduced bioavailability, cellular uptake, and higher anti‐PEG antibody activation [11, 30, 31].

Zeta potential can provide additional insights for LNP formulations, as strong electrochemical charges (∼ ±30 mV) can elevate particle repulsion to improve colloidal stability, but also lead to reduced circulation half‐life and increased cellular toxicity [32, 33]. Zeta potential of tested lPG‐LNPs was between −4.10 (MeOlPG‐*block*‐lPG) and −6.78 mV (MeOlPG), whereas PEGylated LNPs had a zeta potential of −3.30 mV. As all formulations displayed a nearly neutral zeta potential, cellular toxicity due to excess charges is unlikely [34].

**FIGURE 2:**
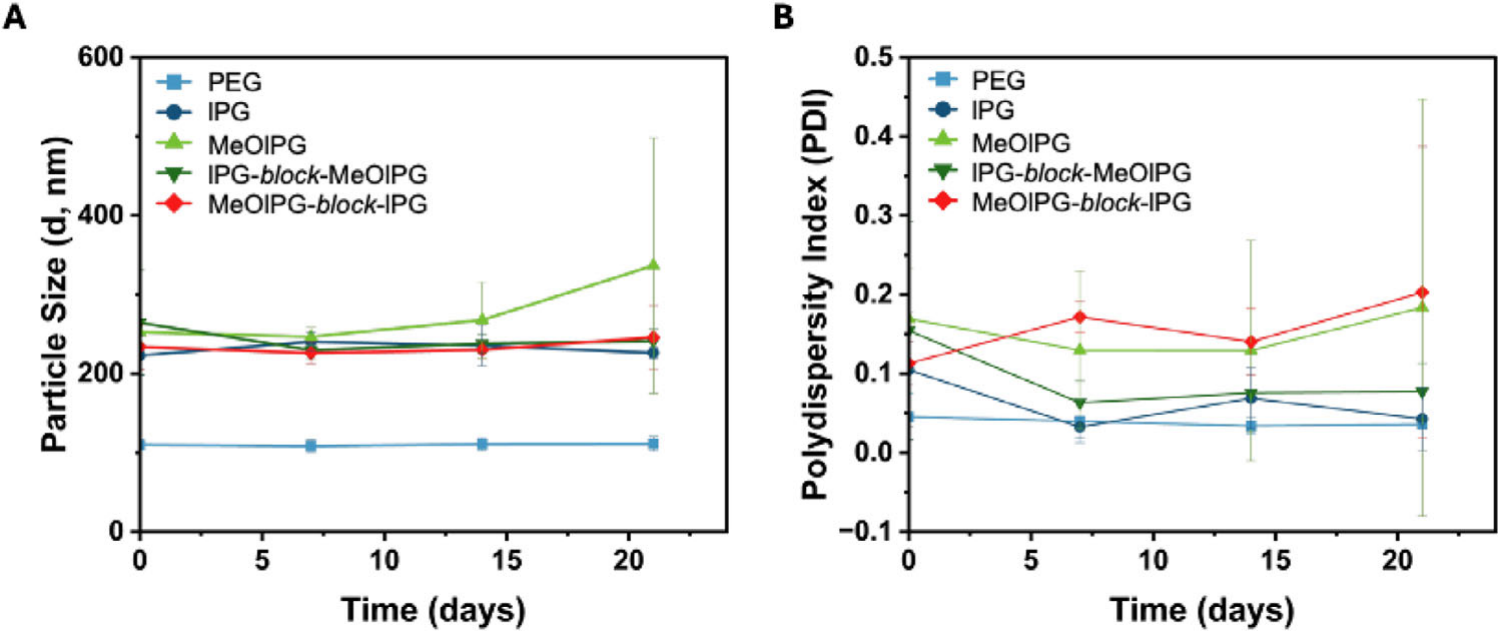
(a) Size and (b) Polydispersity Index over 21 days of mRNA‐LNP systems functionalized with variations of PEG and lPG.

Colloidal storage stability was further assessed by DLS measurements of mRNA‐LNPs at 4°C, demonstrating negligible change in particle diameter over 3 weeks. lPG‐functionalization was able to provide equivalent polymeric stealth effects as PEGylation (Figure 2a, Table S1 Supporting Information). The sole exception was MeOlPG‐LNPs, growing in diameter and PDI during the measurement period (Figure 2b), which may be attributed to MeOlPG's comparatively shorter chain length providing less stealth effects leading to particle agglomeration at 4°C storage [35].

Encapsulation efficiency of mRNA was assessed by comparing unencapsulated (free) mRNA with encapsulated mRNA released after lipid membrane lysis with TritonX‐100. lPG‐LNPs had the highest encapsulation efficiency (93.4%), followed by lPG‐*block*‐MeOlPG (79.4%), MeOlPG‐*block*‐lPG (78.1%), and finally MeOlPG‐LNPs (61.9%). Following, lPG may form compact (albeit slightly larger) LNPs with comparable encapsulation efficiency as PEG‐LNPs (Table 2). While adding some methoxy groups in the studied block‐co‐polymers slightly reduced mRNA encapsulation, MeOlPG had an entirely methoxylated polymer backbone. This feature may contribute to agglomeration and the formation of large, loosely packed MeOlPG‐LNPs with mRNA breaching the particle surface to reduce encapsulation efficiency [31].

#### 2.2.2 Cryo‐TEM Measurements to Assess LNP Morphology

**FIGURE 3:**
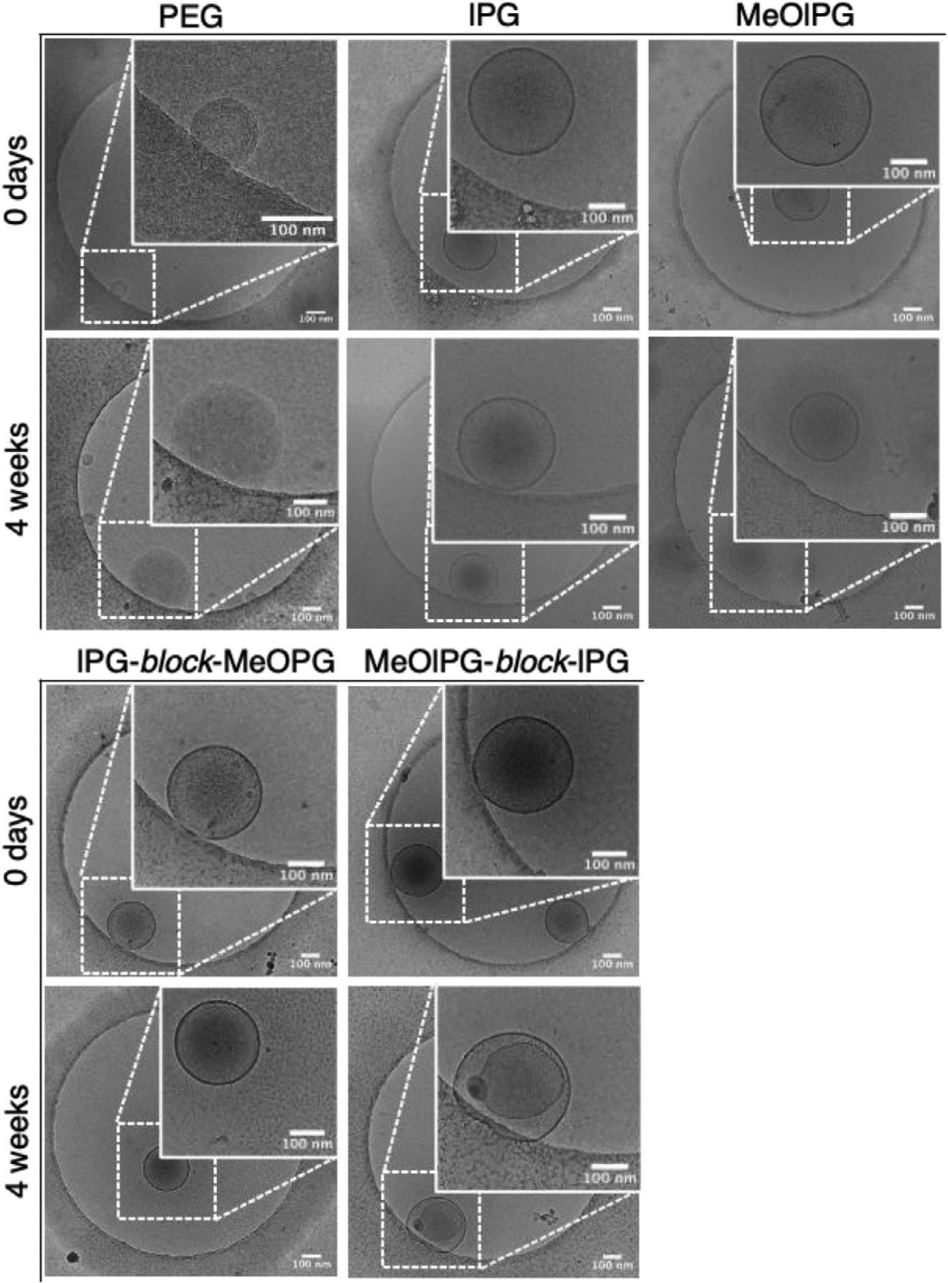
Cryo‐TEM images of PEG‐ and lPG‐functionalized LNPs in PBS immediately after formulation (0 days) and after storage at 4°C for 4 weeks (4 weeks).

Cryo‐TEM imaging of eGFP mRNA LNP systems was performed immediately after microfluidic production (day 0) and after 4 weeks of storage in PBS at 4°C. At day 0, LNPs appeared as unilamellar vesicles in the nanometer range, consistent with prior DLS measurements (Figure 3). As expected, the presence of less definitive lamellar structures was limited due to the relatively high N/P ratio of 6 [36]. Notably, when PEG was replaced with lPG variations, LNP morphology featured a visible lipid bilayer shell rather than a monolayer, surrounding an amorphous electron‐dense core. This “solid core” morphology may result from an interior oil‐phase containing deprotonated ionizable lipids and mRNA [29, 35]. Despite these morphological differences between PEG‐ and lPG‐LNPs, the mRNA encapsulation ability was likely maintained based on previously described results.

After 4 weeks of storage, minor structural changes were observed for all LNP formulations, including thickening of the lipid monolayer and expulsion of mRNA. However, the presence of mainly intact lPG‐LNP systems several weeks after preparation supports previous stability data and may indicate sufficient stability for future therapeutic applications.

#### 2.2.3 Anti‐PEG Antibody Activation

**FIGURE 4:**
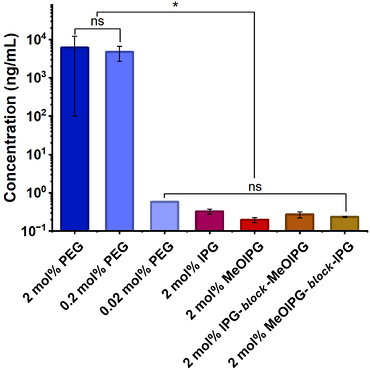
Concentration of PEG (ng mL^−1^) based on anti‐PEG IgG antibody affinity as determined via competitive ELISA for LNPs containing 2 mol% stealth lipid (either PEG or lPG variations) and subsequently diluted to achieve lower concentrations. Statistical significance was evaluated using one‐way ANOVA (* denotes p <0.05; no star denotes lack of significance). Data is representative of 3 independent experiments, as mean ± SD.

A major concern with PEGylated therapeutics is the activation of anti‐PEG antibodies upon administration, leading to accelerated blood clearance (ABC) and hypersensitivity reactions [37, 38]. To observe potential antigenicity and cross‐reactivity, we evaluated the anti‐inflammatory activity of lPG‐LNPs with anti‐PEG antibodies. Previous studies found good agreement between ELISA results using human serum anti‐PEG IgG and commercially available animal anti‐PEG IgG [39]. In the present assay, we used rabbit monoclonal IgG anti‐PEG antibodies, which selectively bind to the terminal methoxy groups of mPEG via Van der Waals and hydrogen bond interactions [40, 41]. Although all lPG‐variations contained terminal methoxy groups, their binding affinity to anti‐PEG antibodies was negligible. ELISA measurements revealed binding concentrations of 0.83 (lPG), 0.50 (MeOlPG), 0.68 (lPG‐*block*‐MeOlPG), and 0.66 ng mL^−1^ (MeOlPG‐*block*‐lPG), respectively (Figure 4). In contrast, PEG‐LNPs at 2 mol% and 0.2 mol% displayed significantly elevated binding activity (1310.55 and 816.81 ng mL^−1^, respectively). A 100‐fold dilution of PEG‐LNPs was required to reduce anti‐PEG antibody binding to levels comparable to those observed for lPG‐LNPs (0.58 ng mL^−1^, *p* < 0.05).

This is a promising result, as mPEG has previously been associated with high anti‐PEG antibody titers compared to other PEG derivatives [22, 42]. Additionally, the C─C─O motif in the PEG backbone is known to promote antibody recognition via hydrophobic interactions, with 3–7 antibody binding sites per monomer [39]. Increased hydrophilicity of the C─C─O unit when substituting PEG with lPG may disrupt such interactions, and remains of interest for future assessments of the antigenicity of lPG‐LNPs [39].

Furthermore, other PEG alternatives such as pOx and pSar have been shown to evade recognition by anti‐PEG antibodies [26, 43]. In line with these findings, our observations show that lPG‐functionalized LNPs may also evade antibody recognition, suggesting a decreased likelihood of ABC effects upon repeat administration [44]. Taken together, these findings further support lPG as a promising PEG alternative for mRNA delivery.

#### 2.2.4 Cell Viability of HepG2 Cells

**FIGURE 5:**
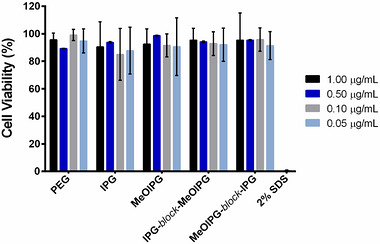
Relative cell viability as determined via CCK8‐assay using HepG2 cells exposed to a variety of amphiphilic polymers derived from lPG, subsequently formulated into LNPs encapsulating mRNA, administered at mRNA‐dosages of 1, 0.5, 0.1, and 0.05 µg mL^−1^ mRNA. Data shows mean ± SD of 5 independent experiments.

To evaluate lPG‐LNPs for gene delivery applications, cell viability of HepG2 cells co‐incubated with mRNA lPG‐LNP systems was assessed in vitro using a CCK8‐assay (Figure 5). As lipid concentrations potentially deviated across formulations, cell viability was assessed using the mRNA‐concentration of each sample. This approach also facilitated consistency with subsequent transfection experiments, which were performed using equivalent mRNA doses. Given the near‐neutral zeta potential of each LNP formulation as well as the known biocompatibility of lPG, minimal cytotoxicity was expected [45, 46]. Cell viability was over 80% when co‐incubated with mRNA concentrations up to 1 µg mL^−1^ in vitro, indicating non‐cytotoxic behaviour at the appropriate doses. Specifically, the highest cell viability at 1 µg mL^−1^ mRNA was observed for lPG‐*block*‐MeOlPG LNPs (95.4%), comparable to PEG‐LNPs (95.6%). The lowest cell viability at 1 µg mL^−1^ mRNA was associated with lPG‐LNPs (90.4%) and overall by MeOlPG‐LNPs (83.4% at 0.5 µg mL^−1^). However, all observed differences across formulations and concentrations were statistically insignificant. These results suggest that modifications of the lPG backbone, such as increased polarity or variations in molecular weight, seemingly had a negligible effect on cell viability.

#### 2.2.5 Transfection of eGFP mRNA Delivered to HepG2 Cells

**FIGURE 6:**
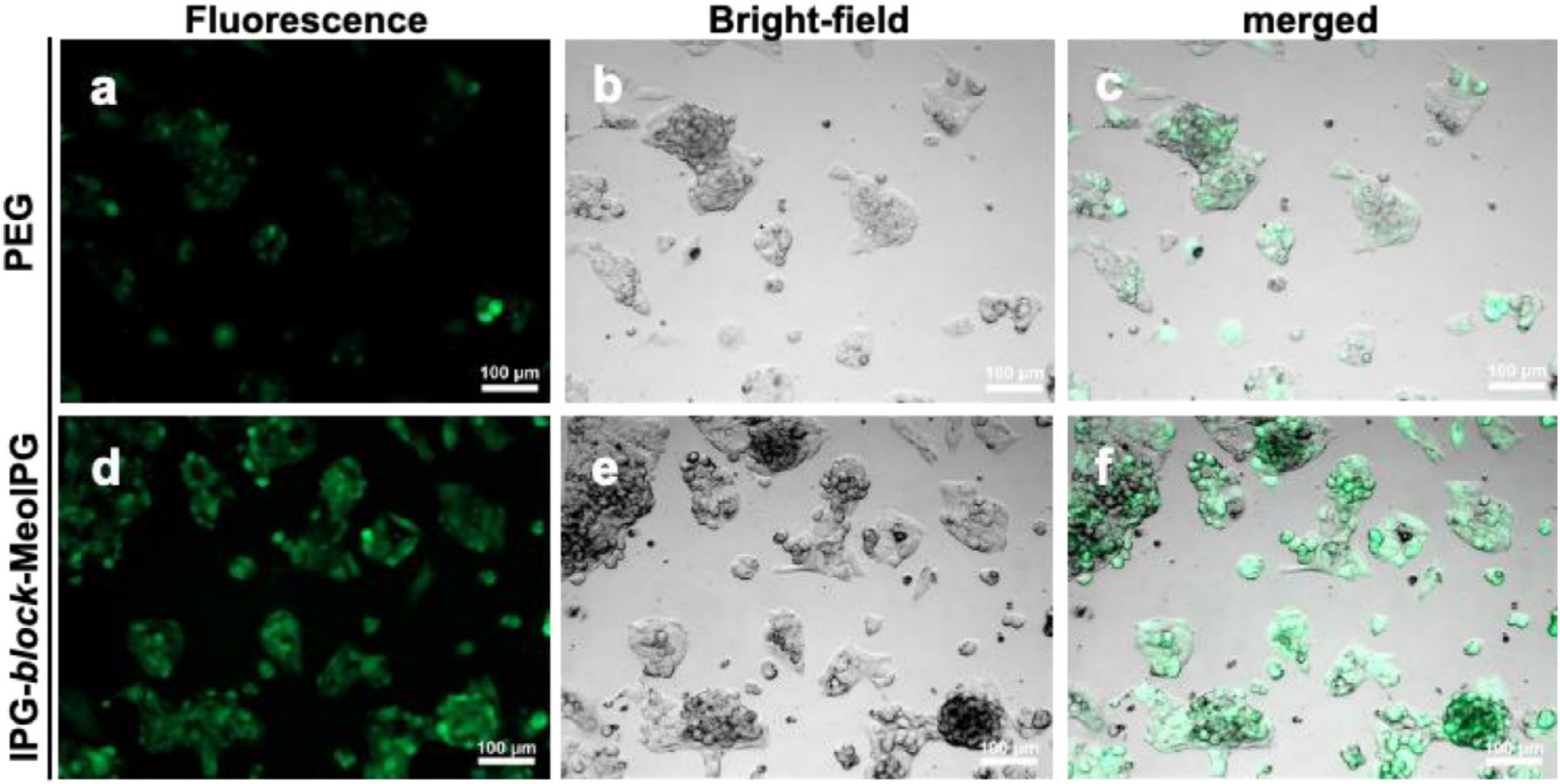
Fluorescence, brightfield, and merged microscope images of HepG2 cells transfected with eGFP mRNA encapsulated in (a–c) PEG‐ and (d–f) lPG‐*block*‐MeOlPG‐functionalized LNPs after 24 h.

As cellular uptake and transfection efficiency have previously been described as dependent on PEG chain length or degree of PEGylation, we sought to investigate if similar factors in lPG‐LNPs influence delivery and intracellular mRNA release [30]. To model mRNA delivery, HepG2 cells were transfected with lPG‐LNPs encapsulating eGFP‐encoding mRNA. Subsequent fluorescence microscopy showed successful cellular uptake and translation of mRNA to express eGFP throughout cells (Figure 6a–f). These results indicated transfection ability was maintained when replacing PEG‐ with lPG‐variations in LNP formulations.

**FIGURE 7:**
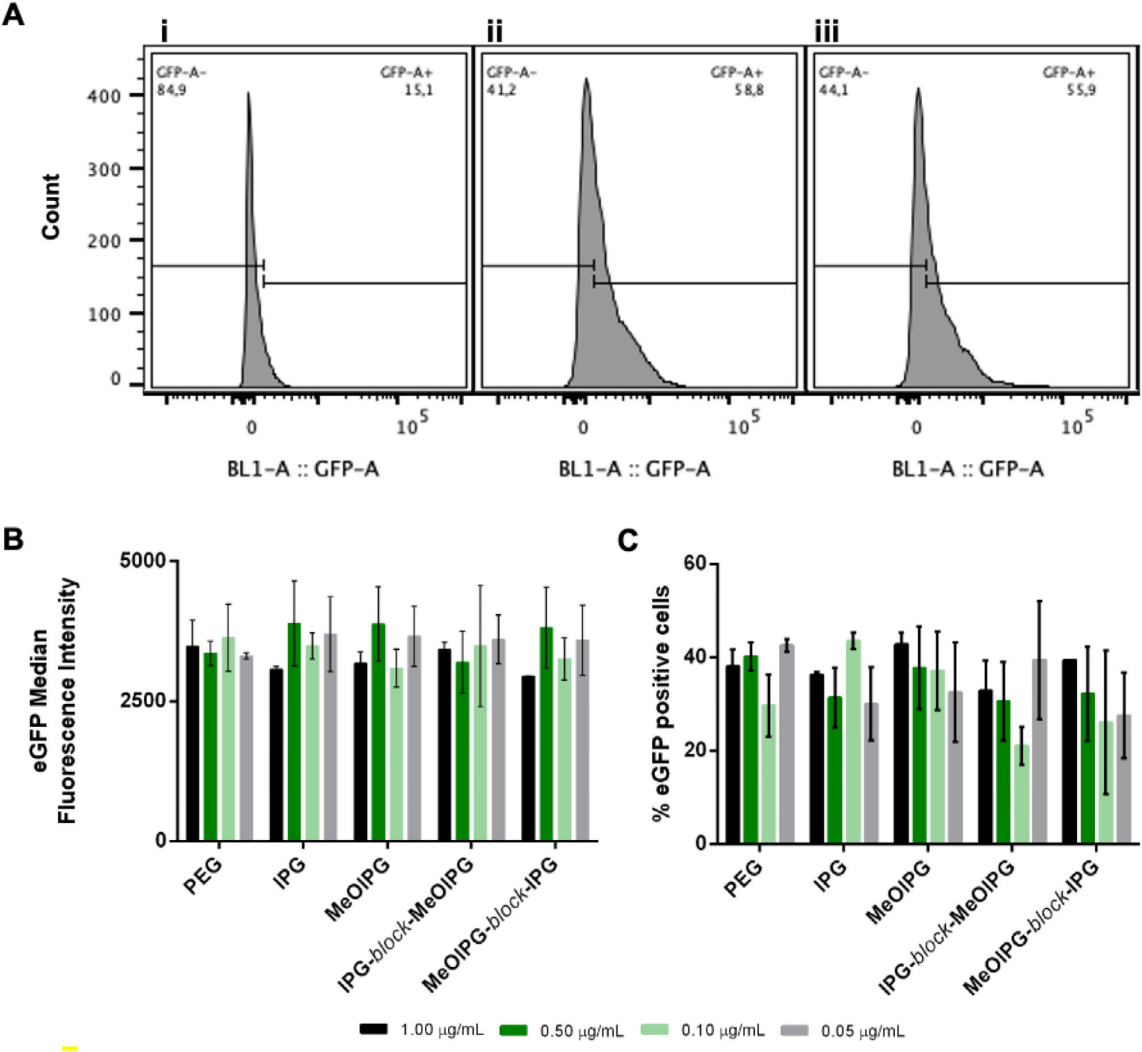
(a) Histograms of cells co‐incubated with (i) media (control), or eGFP mRNA encapsulated in (ii) PEG‐ and (iii) lPG‐LNP systems; Analysis of eGFP fluorescence rendered the following graphs, showing (b) eGFP fluorescence intensity in individual HepG2 cells after transfection with mRNA‐LNP systems; and (c) % of eGFP positive cells per cell population. Data displays (b) median ± SD and (c) mean ± SD of 4 independent experiments.

As HepG2 cells tend to aggregate and form clusters, a more detailed analysis was obtained by flow cytometry to quantify fluorescence of individual cells, as well as the proportion of eGFP‐expressing cells (Figure 7a). Median fluorescence intensity per cell (Figure 7b) ranged from 3068.0 to 3890.0 across all lPG‐ and PEG‐LNPs alike, independent of polymer length, functionalization, and mRNA dosage. Results indicate effective intracellular delivery and release of mRNA with all tested lPG‐variations, even at concentrations as low as 0.05 µg mL^−1^ (corresponding to approximately 0.05 pg mRNA per cell for seeding densities of 10 000 cells per well). Previous studies suggest that secondary amine end groups, such as those found in pSar‐functionalized LNPs, can provide a slight positive charge to interact with the negatively charged cellular membrane and enhance mRNA delivery [28]. Given the highly functionalizable backbone of lPG, introducing similarly charged moieties may offer a promising strategy to improve cellular uptake.

While all LNP systems successfully delivered mRNA, delivery efficiency varied between formulations (Figure 7c). Around 43% of cells treated with lPG‐ or MeOlPG‐LNPs exhibited eGFP fluorescence. A dose‐dependent response was observed, with eGFP expression decreasing to around 30% at lower mRNA concentrations. Differences in methylation and side‐group polarity seemingly had a negligible impact, as indicated by the insignificant difference between these formulations. Notably, transfection efficiency was not impacted by the lower initial encapsulation efficiencies and increased particle diameters of MeOlPG‐LNPs.

Interestingly, adding block‐co‐polymers to LNPs slightly reduced transfection efficiency to around 40% at higher mRNA concentrations and around 25% at lower concentrations. Reduced transfection efficiency over 24 h may result from slower transfection rates when using block‐co‐polymers rather than homopolymers in LNP formulations. Although previous studies suggested a dependence on polymer chain length for transfection efficiency, such an effect was not observed—likely due to the modest difference in repeating units of the polymers tested (42 for lPG vs. 27 for MeOlPG), which may be insufficient to significantly influence cellular uptake [25].

## 3 Conclusion

Our study successfully established different linear polyglycerol (lPG) as biocompatible, hydrophilic polymers that can successfully form amphiphilic structures when covalently linked to alkyl chains. These lPG‐amphiphiles can be used as appropriate alternatives to the commercial standard PEG in LNP formulations for mRNA delivery, with comparable transfection efficiency. The most promising candidate as a PEG alternative in LNP formulations was linear polyglycerol (lPG), possessing a similar chain length as PEG2000. While able to provide LNPs with similar size, stability, and transfection efficiency, lPG had negligible binding of anti‐PEG antibodies. In comparison, MeOlPG, with a significantly shorter chain length and methoxylated polymer backbone resulted in similar antibody binding, but due to low mRNA loading, required much larger volumes for similar transfection efficiency. Overall, novel PG‐based lipids show a stable transfection of cells with mRNA and low cross‐reactivity with anti‐PEG antibodies.

## 4 Experimental Section

### 4.1 Materials and Analytical Methods

All commercially available compounds required for polymer synthesis were purchased from Sigma–Aldrich or TCI. To monitor reaction progress, pre‐coated thin‐layer chromatography (TLC) plate (Merck silica gel 60F254) was used, visualizing spots on the TLC plate using potassium permanganate solution. NMR spectra were measured using ECP500 (500 MHz) from JEOL and Avance 500 (500 MHz) from Bruker. Gel permeation chromatography (GPC) was performed in tetrahydrofuran using a SECcurity 1200 from Agilent.

For LNP formulation, 1,2‐distearoyl‐sn‐glycero‐3‐phosphocholine (DSPC, CAS: 816‐94‐4) and 1,2‐dimyristoyl‐rac‐glycero‐3‐methoxypolyethylene glycol‐2000 (DMG‐PEG2000, CAS: 160743‐62‐4) were purchased from Avanti Polar Lipids. LP‐01 (CAS: 1799316‐64‐5) was purchased from WuXi Biologics (WuxiCity, China). Poly(A) mRNA was purchased from Roche, whereas Reporter eGFP mRNA was purchased from TriLink Biotechnologies (L‐7601). RNA concentrations were quantified using a Quant‐iT Ribogreen RNA Assay (ThermoFisher,). Competitive enzyme‐linked immunosorbent assay (ELISA) kits were purchased from Abcam (ab215546). For cell work, high‐glucose Dulbecco's Modified Eagle Medium (DMEM) with GlutaMAX supplement, as well as Dulbecco's Phosphate Buffered Saline (DPBS) was purchased (Gibco, ThermoFisher). Cell counting kit‐8 (CCK‐8) was purchased from Sigma–Aldrich (#96992, Merck).

### 4.2 Synthesis

For synthetic purposes, we followed anionic ring‐opening polymerization using *rac*‐2,3‐bis(benzyloxy)propan‐1‐ol as initiator via reaction with the respective oxirane monomer. The four synthesized polymeric lipids were then formulated into LNPs using microfluidic techniques.

#### 4.2.1 General Procedure for Anionic Ring‐Opening Polymerization for Homopolymer Synthesis

The linear polyglycerol derivatives (Poly(ethoxy ethyl glycidyl ether)s (PEEGE) as precursors for both lPGs, and MeOlPGs) were prepared by living anionic polymerization with *rac‐*2,3‐bis(benzyloxy)propan‐1‐ol **1** as initiator via reaction with the respective oxirane monomer. Initiator **1** (0.25 g, 0.92 mmol) was dissolved in KO*t*Bu in THF (0.20 g, 1.8 mmol in 5 mL THF) under argon atmosphere and heated to 50°C for 2 h to deprotonate the alcohol group. The generated *t*‐BuOH and solvent were removed in high vacuum. The remaining alcoholate initiator was completely dried, re‐dissolved in dry toluene, and heated to 80°C under argon atmosphere. Then, freshly distilled monomer was completely dried (either *rac‐*ethoxyethyl glycidyl ether (EEGE, **2**, 3.0 mL, 21 mmol) for PEEGE, or *rac‐*glycidyl methyl ether (**3**, 1.6 mL, 18 mmol) for MeOlPG), was added to the alcoholate toluene solution and polymerized for 24 h at 80°C under argon atmosphere. The reaction was quenched by first cooling down to rt, followed by the addition of methyl iodide (1 mL) and further stirring at RT for 2 h. After this time, the mixture was concentrated under reduced pressure, and subsequently dried in vacuum. For purification, the obtained oil was dissolved in Et_2_O and centrifuged to separate the insoluble salts. Further purification of the polymer was done by flash chromatography followed by dialysis in acetone (1 kDa MWCO, RC tubing) for 3 days. The dibenzyl‐functionalized PEEGE **2a** and MeOlPG **3a** were obtained as slightly yellow oils with 80% yield (Scheme 1).

**Compound 2a** ^1^H‐NMR: (500 MHz, CDCl_3_, TMS): δ (ppm) = 1.19 (t, J = 7.04 Hz 128H, OCHC*H_3_*), 1.28 (d, J = 5.24 Hz, 128H, CHC*H_3_*), 3.74‐3.38 (m, 307H, *H_a_, H_b_*, *H_c_*, OC*H_2_* CH_3_), 4.52 (s, 2H, CH_f_), 4.72‐4.5 (m, 42H, CH_e_, OC*H*C*H_3_*) 7.34‐7.26 (m, 10H, Bn). M_n_(NMR): 6676 g/mol, n = 42.

**Compound 3a** 1H‐NMR: (500 MHz, CDCl_3_, TMS): δ (ppm) = 3.33 (s, 82H, OC*H_3_*), 3.75‐3.36 (m, 140H, CHC*H_3_*), 4.52 (s, 2H, CH_f_), 4.66 (s, 2H, CH_e_) 7.33‐7.26 (m, 10H, Bn). M_n_(NMR): 2920 g/mol, n = 27.

#### 4.2.2 General Procedure of Anionic Ring‐Opening Polymerization for Copolymer Synthesis

The copolymerized polyglycerols (consisting of lPG and MeOlPG), were prepared by living anionic polymerization with *rac‐*2, 3‐bis(benzyloxy)propan‐1‐ol **1** as the initiator via reaction with the respective oxirane monomer. Initiator **1** (0.25 g, 0.92 mmol) was dissolved in 1 M KOtBu in THF (8.2 mL, 8.2 mmol) under argon atmosphere and heated to 50°C for 2 h. The generated *t*‐BuOH and solvent were removed in a vacuum. The remaining alcoholate initiator was completely dried, re‐dissolved in dry toluene, and heated to 80°C under an argon atmosphere. For copolymer **4a** [Bn_2_O‐PEEGE‐*b*‐MeOlPG‐OCH_3_], freshly distilled monomer ethoxyethyl glycidyl ether (2.0 mL, 14 mmol) was added to the mixture and stirred for 12 h, followed by glycidyl methyl ether (1.0 mL, 12 mmol) for the second polymer unit of MeOlPG and further stirring for 24 h at 80°C under argon atmosphere. Similarly, for block copolymer **5a** [Bn_2_O‐MeOlPG‐*b*‐PEEGE‐OCH_3_], glycidyl methyl ether (1.0 mL, 12 mmol) was added to the initiator alcoholate and stirred for 12 h, followed by the addition of ethoxyethyl glycidyl ether (2.0 mL, 14 mmol) for the second polymer unit of PEEGE to polymerize for a further 24 h at 80°C under argon atmosphere.

The reaction was quenched by the reaction of methyl iodide (1 mL), similarly to the synthesis of homopolymers. The mixture was left stirring for 1–2 h at rt, then concentrated under reduced pressure and subsequently dried in vacuum. For purification, the obtained oil was dissolved in Et_2_O and centrifuged to separate the insoluble salts. Further purification of the polymer was done by flash chromatography and dialysis in acetone (1 kDa MWCO, RC tubing) for 3 days. The dibenzyl‐functionalized block copolymers **4a** and **5a** were obtained as slightly yellow oils (Scheme 2).

**Compound 4a** ^1^H‐NMR: (500 MHz, CDCl_3_, TMS): δ (ppm) = 1.19 (t, J = 7.04 Hz 54H, OR_1_(OCHCH_3_)), 1.28 (m, 54H, CHC*H_3_*), 3.33 (s, 40H, OR_2_(OCH_3_)), 3.70‐3.37 (m, 220H, *H_a_, H_b_*, *H_c_*, *H_d_, H_e_*, *H_f_* OR_1_(OC*H_2_* CH_3_)), 4.52 (s, 2H, CH_f_), 4.70‐4.52 (m, 20H, CH_g_, OC*H*C*H_3_*), 7.34‐7.26 (m, 10H, Ar). M_NMR_ : 4316, n_(EEGE)_ = 18, m_(OMe)_ = 13.

**Compound 5a** ^1^H‐NMR: (500 MHz, (CD_3_)_2_CO, TMS): δ (ppm) = 1.19 (t, J = 7.04 Hz 68H, OCHC*H_3_*), 1.28 (m, 68H, CHC*H_3_*), 3.33(s, 58H, OR_1_(OCH_3_)), 3.70‐3.37 (m, 271H, *H_a_, H_b_*, *H_c_*, *H_d_, H_e_*, *H_f_* OR_2_(OC*H_2_* CH_3_)), 4.50 (s, 2H, CH_f_), 4.66‐4.50 (m, 20H, CH_g_, OC*H*C*H_3_*), 7.34‐7.26 (m, 10H, Ar). M_NMR_: 5429, n_(OMe)_ = 19, m_(EEGE)_ = 22.

#### 4.2.3 General Procedure of Hydrogenative Debenzylation

The lPG dibenzyl ethers were deprotected via hydrogenation in Toluene with Pd/C (10% w/w) as a catalyst. Compounds **2a, 3a, 4a**, and **5a** were each dissolved in Toluene and flushed with argon three times. Then hydrogen gas was flushed into the solution and the reaction was left in hydrogen atmosphere at room temperature for 3–4 days. The mixture was filtered through Celite to remove the catalyst, and the filtrate was concentrated under reduced pressure. The diol‐functionalized (Polymer‐OCH_2_CH(*OH*)CH_2_ *OH*) were obtained as colorless, viscous oils in quantitative yield after drying in high vacuum.

**Compound 2b** ^1^H‐NMR: (500 MHz, CDCl_3_, TMS) 1.19 (t, 3H, OCHC*H_3_*,), 1.28 (d, 3H, CHC*H_3_*), 3.74‐3.38 (m, 7H, *H_a_, H_b_*, *H_c_*, OC*H_2_* CH_3_), 4.72‐4.5 (m, 1H, CH_e_, OC*H*C*H_3_*)

**Compound 3b** ^1^H‐NMR: (500 MHz, CDCl_3_, TMS): δ (ppm): 3.32 (s, 3H, OCH_3_,) 3.67‐3.34(m, 5H, *H_a_, H_b_*, *H_c_*,)

**Compound 4b** ^1^H‐NMR: (500 MHz, (CD_3_)_2_CO, TMS): δ (ppm) 1.19 (m, 3H, OR_1_(OCHCH_3_)), 1.23 (m, 3H, OR_1_(CHC*H_3_*), 3.33(s, 3H, OR_2_(OCH_3_)), 3.70‐3.37 (m, 12H,*H_a_, H_b_*, *H_c_*, *H_d_, H_e_*, *H_f_* OR_1_(OC*H_2_* CH_3_)), 4.70‐4.64 (m, 1H, OC*H*C*H_3_*).

**Compound 5b** ^1^H‐NMR: (500 MHz, CDCl_3_, TMS): δ (ppm) 1.19 (m, 3H, OR_2_(OCHCH_3_)), 1.29 (m, 3H, OR_2_(CHC*H_3_*), 3.33(s, 3H, OR_2_(OCH_3_)), 3.74‐3.39 (m, 12H,*H_a_, H_b_*, *H_c_*, *H_d_, H_e_*, *H_f_* OR_1_(OC*H_2_* CH_3_)), 4.70‐4.64 (m, 1H, OC*H*C*H_3_*).

#### 4.2.4 Synthesis of Double Ester Polymer Lipids

To install the lipid ester moieties, the diol‐functionalized lPG was dissolved in DCM under argon atmosphere and triethylamine (4 equiv.) was added at room temperature. The reaction mixture was stirred for 30 min and then cooled down to 0°C, upon which myristoyl chloride (2.4 equiv) was added dropwise. The mixture was stirred for 2 days at room temperature and then purified by flash column chromatography followed by dialysis in regenerated cellulose membrane with 1 kDa MWCO in acetone to yield the diester products. The methoxylated lPG amphiphile **3c** was obtained as colorless oil (80% yield).

**Compound 2c** ^1^H‐NMR: (500 MHz, CDCl_3_, TMS): δ (ppm) = 0.88‐0.86 (t, 6H, C*H_3_*), 1.19 (t, m, 127H, OCHC*H_3_*), 1.28 (m, 168H, CHC*H_3_*, alkyl chain), 1.63‐1.55 (m, CH_e,_ CH_e″_), 2.32‐2.25 (m, CH_d,_ CH_d″_), 3.69‐3.42 (m, 307H,*H_a_, H_b_*, *H_c_*, OC*H_2_* CH_3_), 4.72‐4.5 (m, 42H, OC*H*C*H_3_*).

**Compound 3c** ^1^H‐NMR: (500 MHz, CDCl_3_, TMS): δ (ppm) = 0.88‐0.86 (t, 6H, C*H_3_*), 1.31‐1.27 (m, 40H, alkyl chain), 1.72‐1.54 (m, CH_e,_ CH_e″_), 2.35‐2.25 (m, CH_d,_ CH_d″_), 3.32 (s, 81H, OCH_3_) 3.67‐3.34 (m, 140H, *H_a_, H_b_*, *H_c_*,). M_n_(NMR) = 2860 g/mol.

**Compound 4c** ^1^H‐NMR: (500 MHz, (CD_3_)_2_CO, TMS): δ (ppm) = 0.88‐0.86 (t, 6H, C*H_3_*), 1.17 (m, 56H, OR_1_(OCHC*H_3_*)), 1.28 (m, 110H, OR_1_(CHC*H_3_)* alkyl chain), 1.66‐1.56 (m, CH_g,_ CH_g″_), 2.39‐2.24 (m, CH_h,_ CH_h″_), 3.32 (s, 38H, OR_2_(OCH_3_), 3.67‐3.40 (m, 203H, *H_a_, H_b_*, *H_c_*, *H_d_, H_e_*, *H_f_* OR_1_(OC*H_2_* CH_3_)), 4.73‐4.69 (m, 18H, OR_1_(OC*H*C*H_3_)*).

**Compound 5c** ^1^H‐NMR: (500 MHz, CDCl_3_, TMS): δ (ppm) = 0.88‐0.86 (t, 6H, C*H_3_*), 1.17 (m, 58H, OR_2_(OCHC*H_3_*)), 1.28 (m, 98H, OR_2_(CHC*H_3_)* alkyl chain), 1.63‐1.52 (m, CH_g,_ CH_g″_), 2.34‐2.23 (m, CH_h,_ CH_h″_), 3.34 (s, 57H, OR_1_(OCH_3_)), 3.67‐3.40 (m, 278H, *H_a_, H_b_*, *H_c_*, *H_d_, H_e_*, *H_f_* OR_2_(OC*H_2_* CH_3_)), 4.72‐4.67 (m, 19H, OR_1_(OC*H*C*H_3_)*).

#### 4.2.5 General Procedure for Deprotection of Acetal‐Protected Side Groups

To obtain the hydroxylated side chain amphiphiles, the EEGE‐protected side chains were deprotected. To a 0.05 mg mL^−1^ solution of the PEEGE‐based compound in acetone, an aqueous solution of oxalic acid (2 equiv. per repeating unit of EEGE, as a solution in 3:1 acetone:water, to a concentration of 0.15 g oxalic acid mL^−1^ water) was added dropwise under fast stirring at room temperature. After 3.5 h, the reaction was diluted with deionized H_2_O and dialyzed against deionized H_2_O (1 kDa MWCO, RC tubing) until a pH of 6 to 7 was reached. The solvent was removed under reduced pressure, affording the respective product **2d/4d/ 5d**.

**Compound 2d** ^1^H‐NMR: (500 MHz, MeOD, TMS): δ (ppm) = 0.88‐0.86 (t, 6H, C*H_3_*), 1.28 (m, 40H, alkyl chain), 1.63‐1.55 (m, CH_e_, CH_e″_), 2.32‐2.25 (m, CH_d,_ CH_d″_), 3.69‐3.42 (m, 218H, *H_a_, H_b_*, *H_c_*, OC*H_2_* CH_3_). M_n_(NMR) = 3550 g/mol.

**Compound 4d** ^1^H‐NMR: (500 MHz, CD_3_OD, TMS): δ (ppm) = 0.95‐0.93 (t, 6H,C*H_3_*), 1,34‐1.26 (m, 40H, alkyl chain), 1.68‐1.64 (m, CH_g,_ CH_g″_), 2.41‐2.37 (m, CH_h,_ CH_h″_), 3.39 (s, 38H, OR_2_(OCH_3_)), 3.78‐3.47 (m, 167H, *H_a_, H_b_*, *H_c_*, *H_d_, H_e_*, *H_f_*,). M_n_(NMR) = 3060 g/mol.

**Compound 5d** ^1^H‐NMR: (500 MHz, CD_3_OD, TMS): δ (ppm) = 0.89‐0.87 (t, 6H,C*H_3_*), 1,35‐1.28 (m, 40H, alkyl chain), 1.66‐1.52 (m, CH_g,_ CH_g″_), 2.36‐2.33 (m, CH_h,_ CH_h″_), 3.34 (s, 55H, OR_1_(OCH_3_), 3.78‐3.47 (m, 178H, *H_a_, H_b_*, *H_c_*, *H_d_, H_e_*, *H_f_*).

### 4.3 Microfluidic Preparation of LNPs

The ionizable lipid LP‐01, cholesterol, DSPC and one of the polymer lipids (**2d**, **3c**, **4d**, **5d** or DMG‐PEG2000 as a control)) were mixed at a 50:39:9:2 molar ratio in ethanol to obtain a final lipid concentration of 3 mol.

Aqueous solution for LNP formulation consisted of 1 mg mL^−1^ mRNA in sodium citrate (1 mm, pH 6.4), diluted prior to each use with citrate buffer (10 mm, pH 4) to obtain a final N/P ratio of 6. Cell viability, transfection, and cryo‐EM experiments were performed with LNPs prepared with eGFP mRNA, whereas Poly(A) mRNA was used for remaining measurements.

LNPs were prepared by mixing aqueous and ethanol solutions using a NanoAssemblr Ignite Cartridge with NxGen mixing technology on a NanoAssemblr Ignite system (Precision Nanosystems, Vancouver, BC, Canada). Process flow conditions were 12 mL min^−1^ total flowrate at 3:1 aqueous:ethanol solution. Buffer‐exchange was completed by filling formulations in Amicon Ultra‐4 ultrafiltration centrifuge tubes (MWCO 30 kDa) (Millipore). Formulations were centrifuged at 5000 xg for 15 min, after which the supernatant was removed and volume topped up with PBS. Centrifugation was repeated three times, and formulations were stored at 4°C until use.

### 4.4 Characterization of LNPs

#### 4.4.1 Size, Stability, and Zeta‐Potential

The size of LNPs was measured using dynamic light scattering (Zetasizer Ultra, Malvern Panalytical Ltd) in 1x PBS, reported as the z‐average mean particle diameter. Measurements were repeated at 7 day‐intervals for stability over time, with samples stored at 4°C. Zeta‐potential of 1x LNPs in PBS was measured on a Zetasizer Ultra device using folded capillary zeta cell cuvettes (Malvern Panalytical Ltd.).

#### 4.4.2 Encapsulation Efficiency

A Quant‐iT Ribogreen RNA Assay was used to determine mRNA encapsulation efficiency. Briefly, LNPs were diluted 67‐fold in TE‐buffer. Subsequently, lipid membranes were lysed by adding 2% v/v Triton X‐100 to release encapsulated mRNA. Control samples containing unencapsulated mRNA were diluted with equivalent volumes of TE buffer. Samples were incubated for 10 min at 37°C, before diluting 1:1 with Ribogreen solution. Fluorescence was then quantified by a plate reader (Spark, TECAN) (excitation 485 nm; emission 528 nm), and encapsulation efficiency (EE) was calculated following Equation (1):

(1) $$EE\left(\%\right)=\frac{\left[Total\;mRNA\right]-\left[Unencapsulated\;mRNA\right]}{\left[Total\;mRNA\right]}\times 100$$

#### 4.4.3 Cryo‐TEM Imaging

Chloroform‐cleaned 300 mesh R1.2/1.3 holey carbon grids (Quantifoil, MicroTools GmbH, Jena, Germany) were hydrophilized by glow discharging in an EMSCOPE SC500 (10 mA, 60 s) before applying 4 µL aliquots of mRNA‐LNP solution to grids. Samples were vitrified by automatic blotting (blot force ‐11, blot time 3.5 s, wait time 3 s) and subsequent plunge freezing with an FEI Vitrobot Mark IV (ThermoFisher Scientific) using liquid ethane as cryogen. Grids were clipped with copper clip rings and C‐clips to obtain Autogrids. Vitrified samples were transferred to the autoloader of an FEI TALOS ARCTICA electron microscope (Thermo Fischer Scientific), equipped with a high‐brightness field‐emission gun (XFEG) operated at an acceleration voltage of 200 kV. Micrographs were acquired on a FEI Falcon 3 direct electron detector (Thermo Fischer Scientific) at normal magnifications of 3400, 4300, and 28 000, corresponding to calibrated pixel sizes of 31.3, 24.6, and 3.7 Å pixel^−1^, respectively.

#### 4.4.4 Anti‐PEG Antibody ELISA

Binding of LNPs to anti‐PEG antibodies was measured using a competitive ELISA assay according to the manufacturer's instructions. Briefly, LNPs were diluted 1:1 with PEG‐BSA standard solution before transferring 50 µL to the provided 96‐well plate. The plate was incubated on a plate shaker (400 rpm) for 45 min at rt. Afterward, liquid was aspirated out of each well and the plate was washed using wash buffer three times. Subsequently, 100 µL TMB substrate was added to each well, before incubating on a plate shaker (400 rpm) in the dark for 15 min. Finally, 100 µL stop solution was added per well and mixed for 1 min on a plate shaker, before measuring optical density at 450 nm on a Tecan Spark plate reader.

#### 4.4.5 Cell Culture

HepG2 cells (Leibnitz Institute DSMZ – German Collection of Microorganisms and Cell Cultures GmbH) were cultured at 37°C and 5% CO_2_ in high glucose Dulbecco's Modified Eagle Medium with GlutaMAX supplement (#31966‐021, Gibco/Thermo Fisher Scientific) containing 10% v/v fetal bovine serum (#10270‐106, Gibco/Thermo Fisher Scientific) and 1% v/v penicillin‐streptomycin (#P4333‐100ML, Sigma–Aldrich/Merck). Once cells reached 70% confluency, cells were ready for seeding in experiments.

##### 4.4.5.1 Cell Viability

Seeding density in a 96‐well plate was set as 5 000 cells per well, followed by incubating at 37°C and 5% CO_2_ for 24 h. Subsequently, cell culture media was aspirated out of each well, before adding 100 µL of LNP formulations diluted in fresh media. After returning plates to the incubator for 24 h, 10% v/v CCK‐8 solution (Sigma–Aldrich/Merck), was added to each well and incubated for 3 h before quantifying absorbance on a Spark plate reader (wavelength 450 nm). Cell viability was calculated using Equation (2) for five independent experiments in duplicates:

(2) $$\mathrm{Cell}\;\mathrm{Viability}\;=\frac{\mathrm{Absorbanc}e_{\mathrm{Treated}}-\mathrm{Absorbanc}e_{\mathrm{Blank}}}{\mathrm{Absorbanc}e_{\mathrm{Control}}-\;\mathrm{Absorbanc}e_{\mathrm{Blank}}}\times 100$$

##### 4.4.5.2 Cell Transfection – Evaluation by Flow Cytometry

Seeding density was set to 10 000 cells per well in a 96‐well plate, before incubating at 37°C and 5% CO_2_. After 24 h, mRNA‐LNP samples were diluted with cell culture media to achieve 100, 50, 10 and 5 ng mRNA per 100 µL. Resulting solutions were added to wells and incubated for 24 h. After this time, cells were briefly washed with PBS before adding Trypsin and then PBS to wells to obtain cells in suspension. Subsequently, eGFP fluorescence was measured using Flow Cytometry (Attune NxT Flow Cytometer, Invitrogen, ThermoFisher). Quantification of fluorescence intensity and gene transfection efficiency was determined using FlowJo v10.10 software (FlowJo LLC, Ashland, OR, USA), by selecting desired cell populations and determining background fluorescence in negative controls, before applying appropriate gating to remaining samples.

### 4.5 Statistical Analysis

Statistical analysis of results was performed using Prism 10.2.3 (GraphPad, San Diego, CA, USA). One‐way ANOVA was applied to Figure 3. Tukey's multiple comparison test was applied to determine significant differences between groups. Significance levels were designated as *p < 0.05.

## Conflicts of Interest

The authors declare no conflicts of interest.

## Supporting information




**Supporting File**: marc70048‐sup‐0001‐SuppMat.docx.

## Data Availability

Polyglycerol‐based Lipids: A Next‐Generation Alternative to PEG in Lipid Nanoparticles for Advanced Drug Delivery Systems (07 April 2025, Version 3). ChemRxiv: https://doi.org/10.26434/chemrxiv‐2025‐2jf8r‐v3
