# Supplementary material for: Polyglycerol‐Based Lipids: A Next‐Generation Alternative to PEG in Lipid Nanoparticles for Advanced Drug Delivery Systems
Source: Macromol Rapid Commun. 2025 Sep 16;47(14):e00428. doi: 10.1002/marc.202500428 (PMC13384789; doi:10.1002/marc.202500428)
Supplement: Supplementary file 1 — Supporting File: marc70048‐sup‐0001‐SuppMat.docx. [file MARC-47-e00428-s001.docx]

# Polyglycerol-based Lipids: A Next-Generation Alternative to PEG in Lipid Nanoparticles for Advanced Drug Delivery Systems – Supporting Information

Yara Ensminger^1^, Rashmi Rashmi^1^, Michael Karimov^2^, Gideon Nölte^1^, Markus Hafke^2^, Ann-Cathrin Schmitt^1^, David Diaz-Oviedo^3^, Johannes Köbberling^3^, Rainer Haag^1,*^

^1^ Institute for Chemistry and Biochemistry, Freie Universität Berlin, Takustr. 3, 14195 Berlin, Germany

^2^ Chemical and Pharmaceutical Development, Bayer AG, Pharmaceuticals, Müllerstr. 178, 13353 Berlin, Germany

^3^ Drug Discovery Sciences, Bayer AG, Pharmaceuticals, Aprather Weg 18a, 42113 Wuppertal, Germany

^*^Corresponding author email: haag@zedat.fu-berlin.de

**
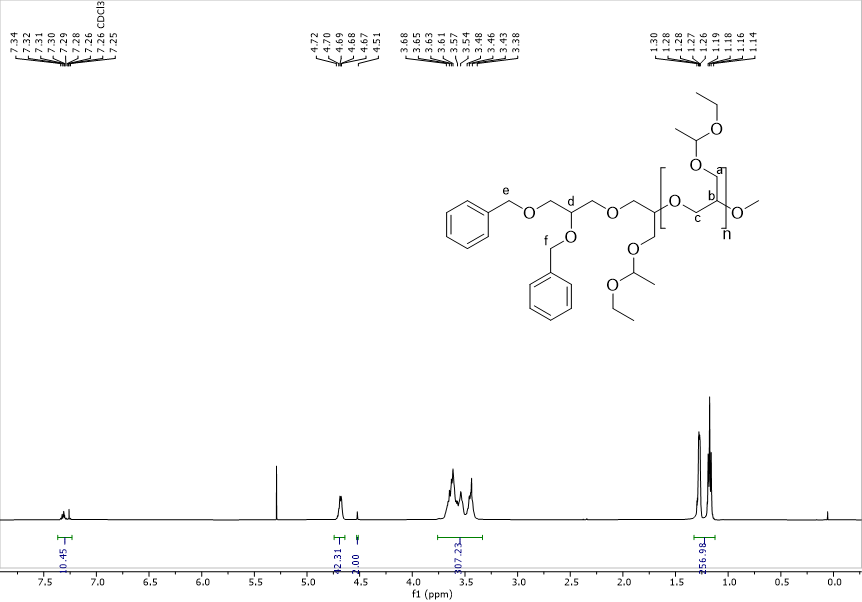
**

**Figure S1:** ^1^H NMR of compound 1a

**
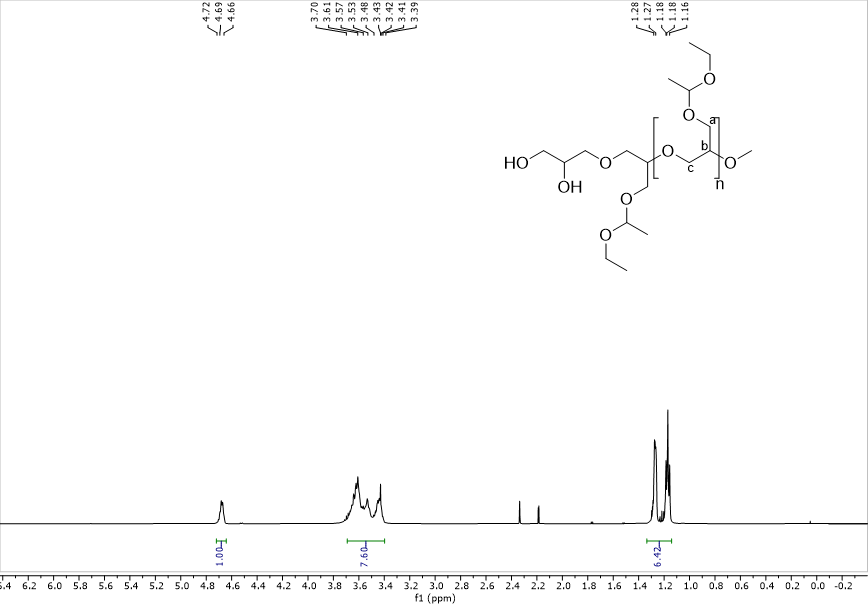
**

**Figure S2:** ^1^H NMR of compound 1b


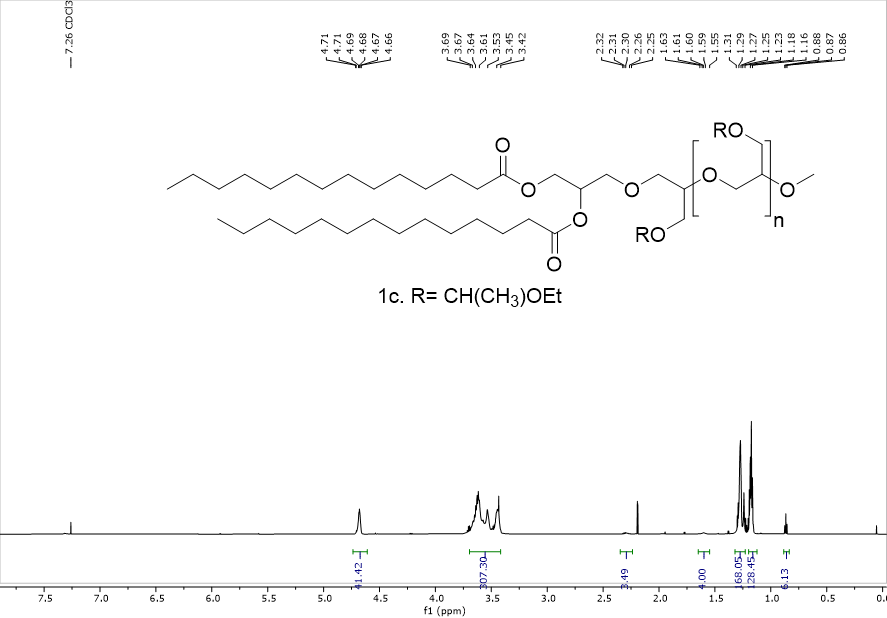


**Figure S3:** ^1^H NMR of compound 1c

**
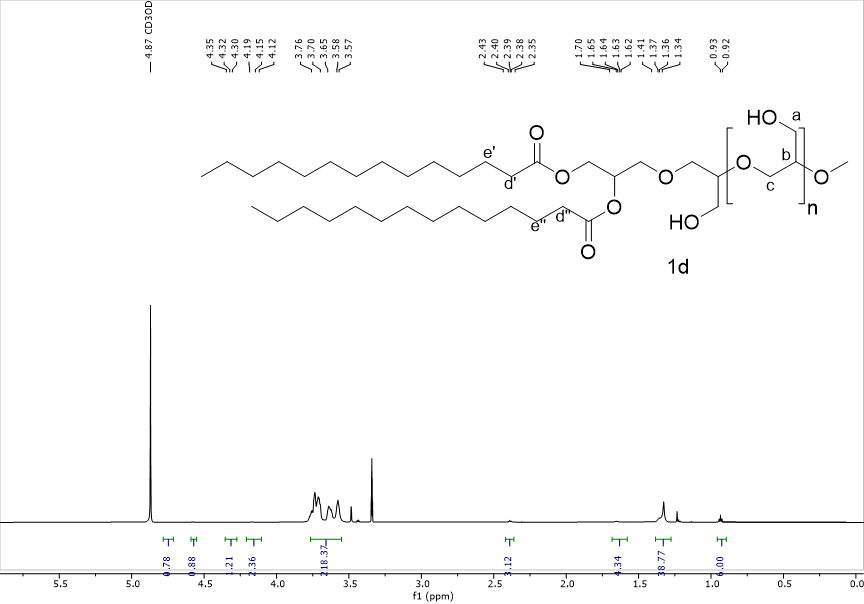
**

**Figure S4:** ^1^H NMR of compound 1d

**
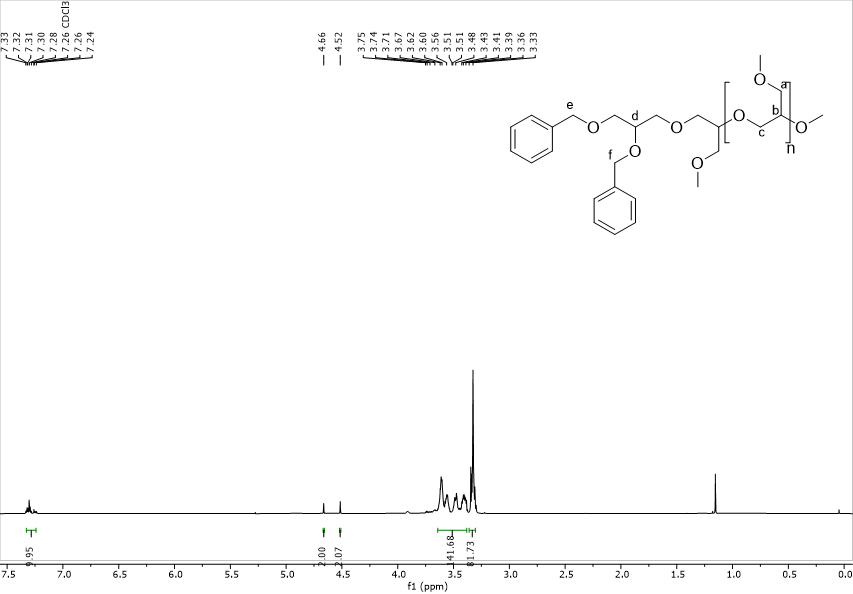
**

**Figure S5:** ^1^H NMR of compound 2a


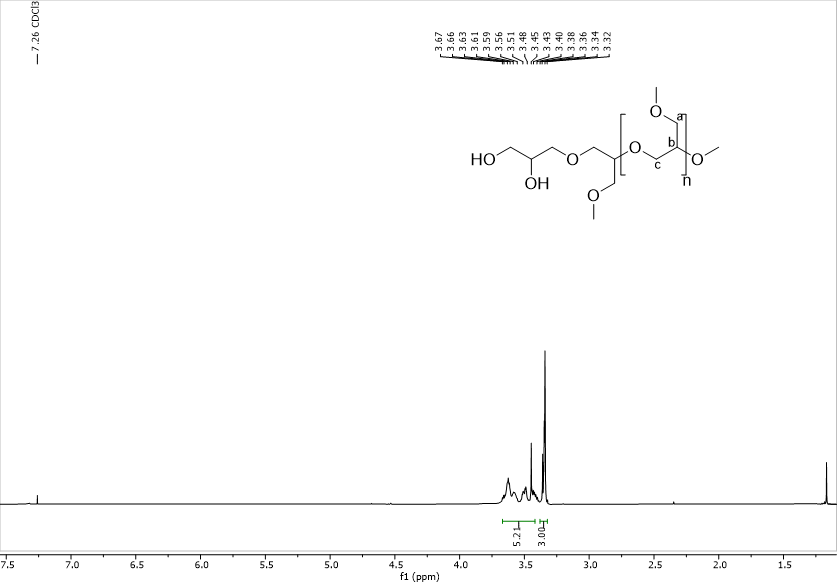


**Figure S6:** ^1^H NMR of compound 2b

**
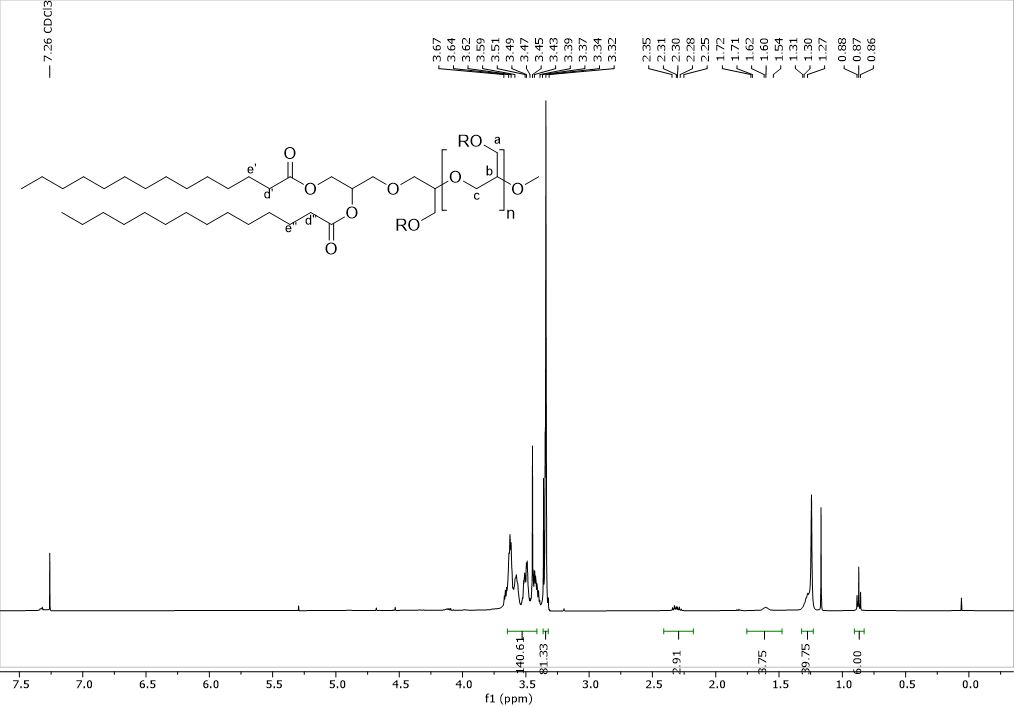
**

**Figure S7:** ^1^H NMR of compound 2c

**
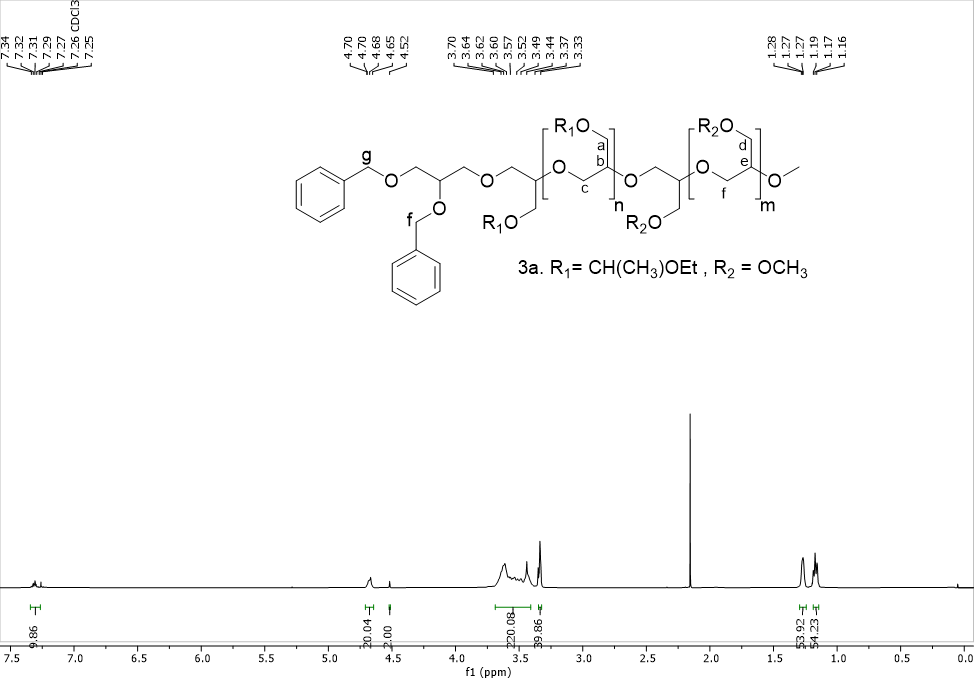
**

**Figure S8:** ^1^H NMR of compound 3a

**
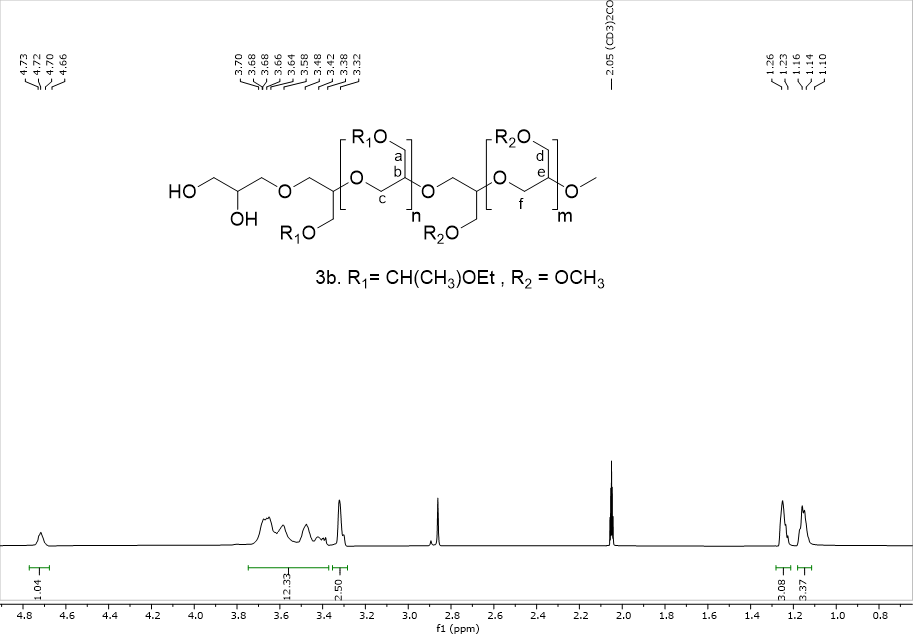
**

**Figure S9:** ^1^H NMR of compound 3b

**
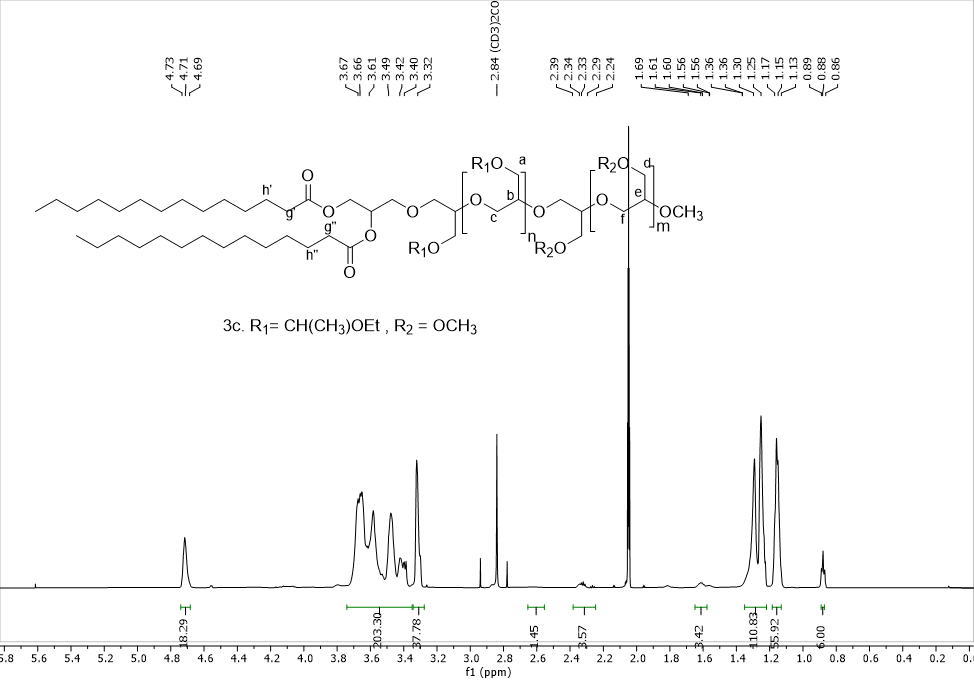
**

**Figure S10:** ^1^H NMR of compound 3c

**
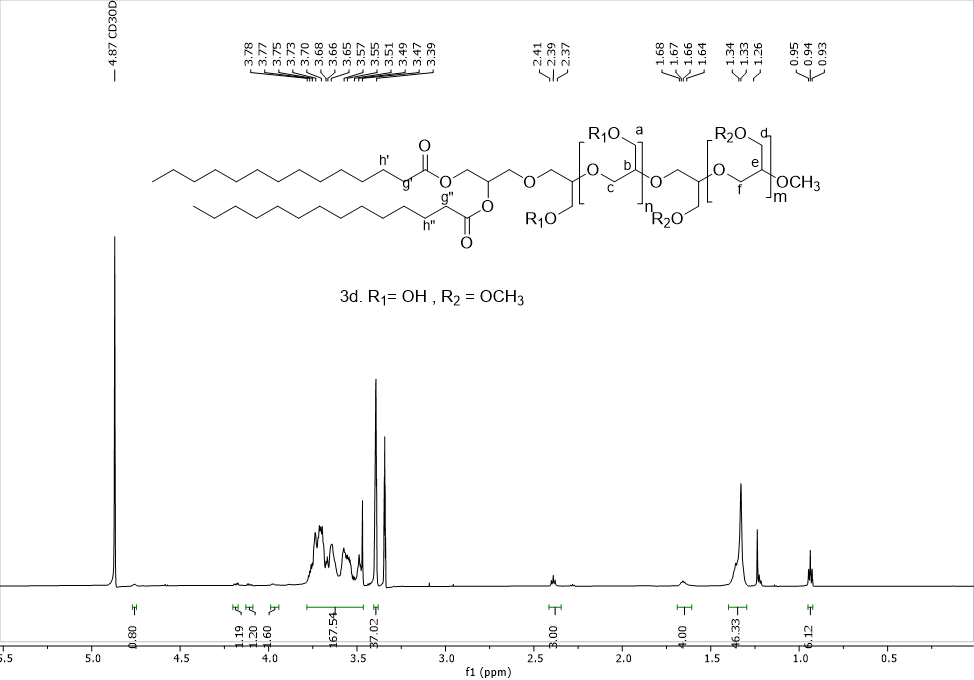
**

**Figure S11:** ^1^H NMR of compound 3d

**
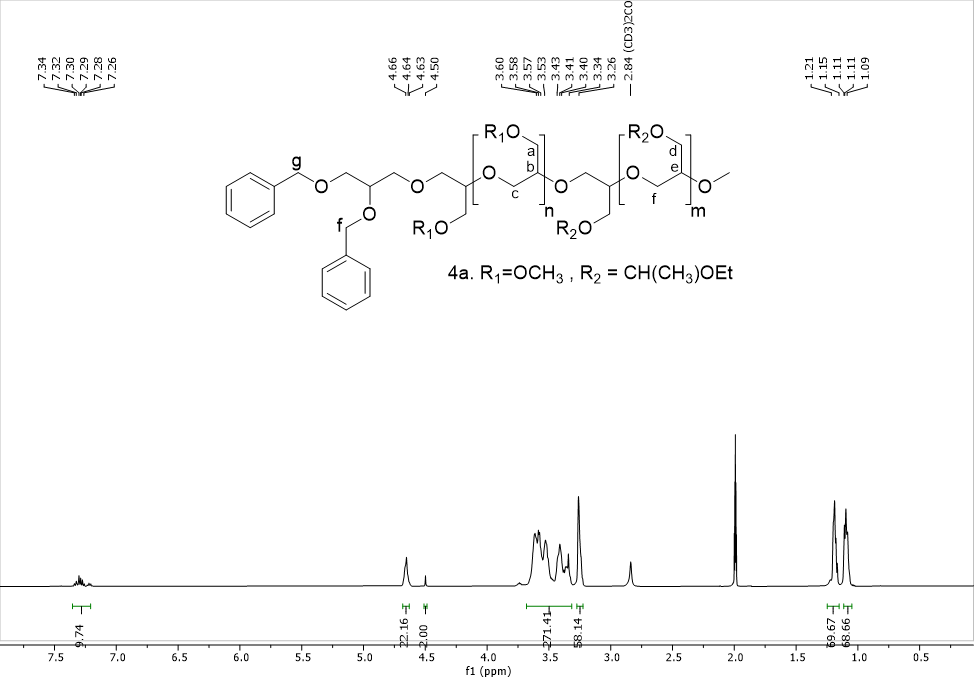
**

**Figure S12:** ^1^H NMR of compound 4a

**
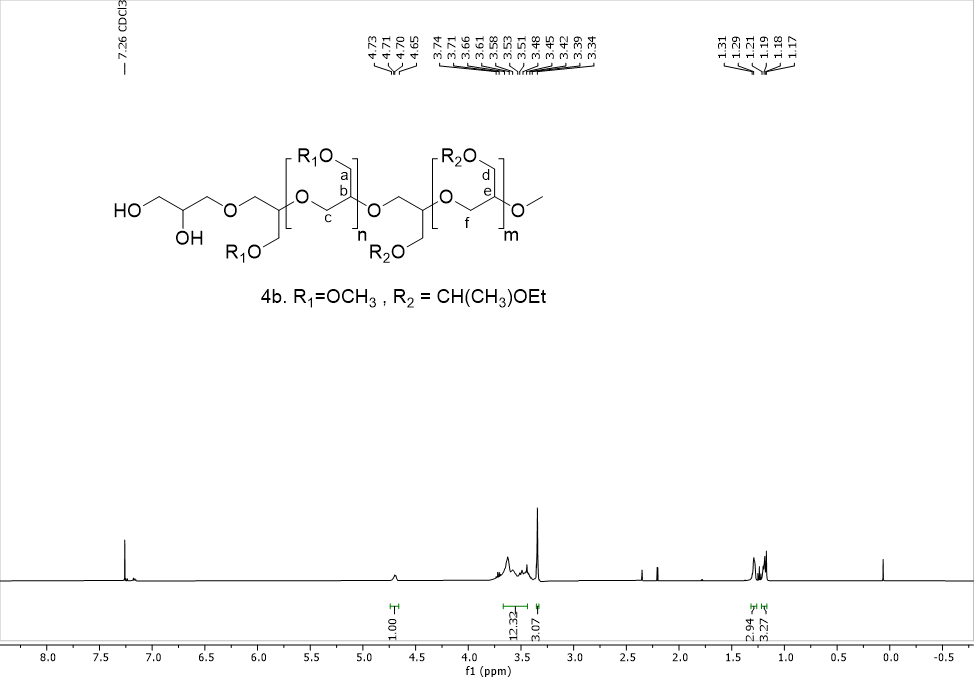
**

**Figure S13:** ^1^H NMR of compound 4b

**
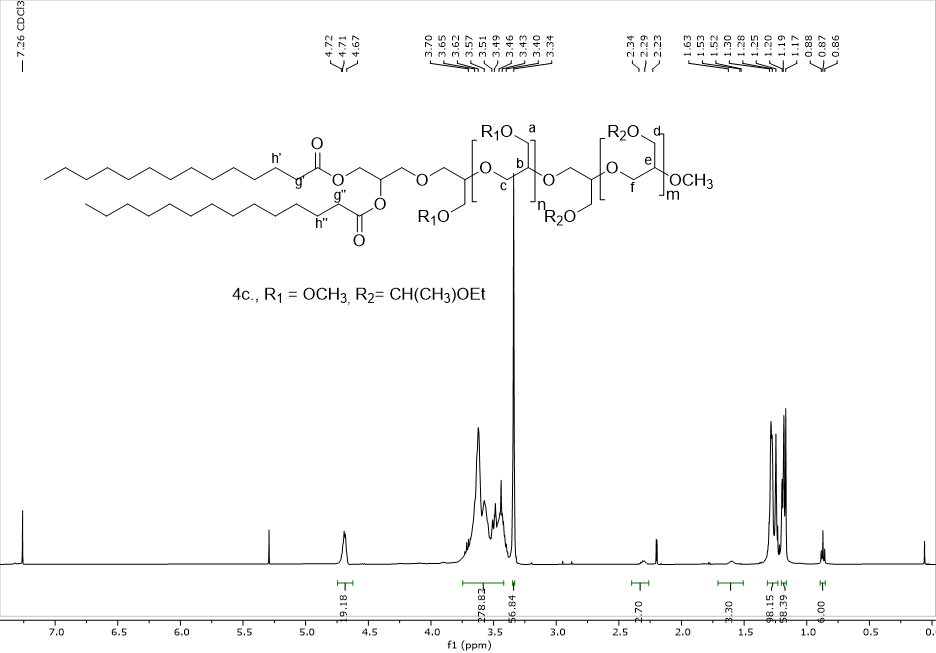
**

**Figure S14:** ^1^H NMR of compound 4c

**
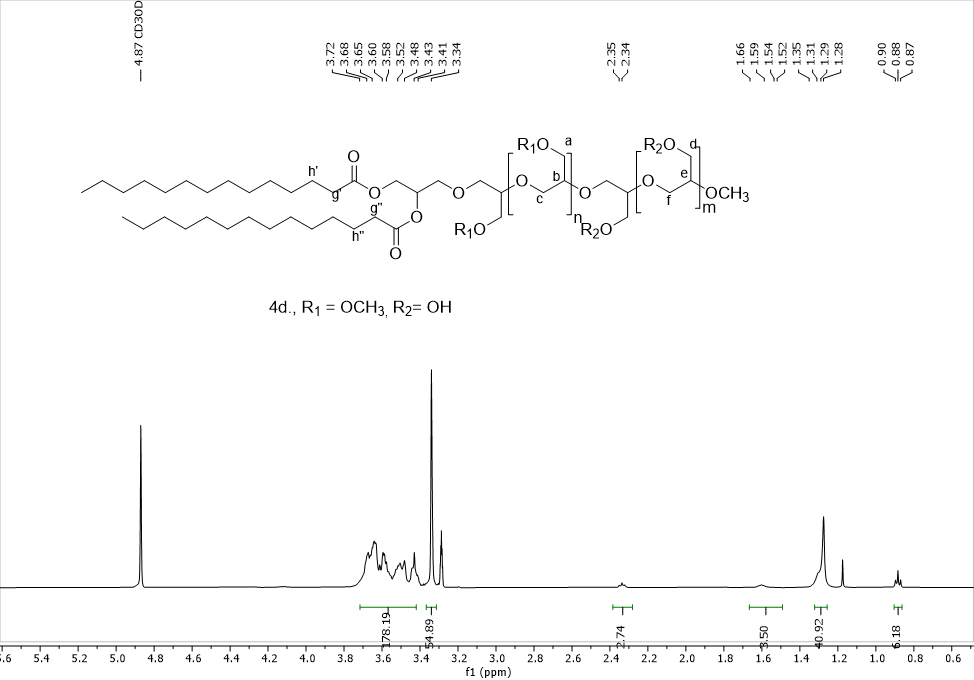
**

**Figure S15:** ^1^H NMR of compound 4d

**Table S1:** Z-average mean particle diameter of PG- and PEG-LNPs over 21 days

| **Time (days)** | **PEG** | **lPG** | **MeOlPG** | **lPG-*b*-MeOlPG** | **MeOlPG-*b*-lPG** |
| --- | --- | --- | --- | --- | --- |
| 0 | 110.0 ± 6.3 | 223.1 ± 24.4 | 252.3 ± 14.0 | 264.3 ± 66.4 | 233.7 ± 28.2 |
| 7 | 108.4 ± 7.9 | 240.3 ± 11.5 | 246.5 ± 11.7 | 229.8 ± 11.5 | 226.2 ± 13.3 |
| 14 | 110.8 ± 7.9 | 235.3 ± 25.5 | 267.7 ± 48.3 | 237.8 ± 11.5 | 230.1 ± 11.0 |
| 21 | 111.2 ± 9.1 | 226.4 ± 6.8 | 336.6 ± 161.8 | 241.0 ± 16.0 | 245.9 ± 39.9 |

**Table S2:** Polydispersity Index of PG- and PEG-LNPs over 21 days

| **Time (days)** | **PEG** | **lPG** | **MeOlPG** | **lPG-*b*-MeOlPG** | **MeOlPG-*b*-lPG** |
| --- | --- | --- | --- | --- | --- |
| 0 | 0.045 ± 0.030 | 0.105 ± 0.072 | 0.169 ± 0.063 | 0.154 ± 0.138 | 0.113 ± 0.027 |
| 7 | 0.040 ± 0.027 | 0.033 ± 0.013 | 0.130 ± 0.100 | 0.063 ± 0.028 | 0.172 ± 0.020 |
| 14 | 0.034 ± 0.011 | 0.069 ± 0.039 | 0.129 ± 0.139 | 0.076 ± 0.049 | 0.140 ± 0.042 |
| 21 | 0.036 ± 0.001 | 0.043 ± 0.040 | 0.183 ± 0.264 | 0.077 ± 0.036 | 0.203 ± 0.184 |

**Table S3:** Anti-PEG antibody affinity to PG- and PEG-LNP systems as determined via competitive ELISA utilizing anti-PEG IgG antibodies to quantify PEG concentration in samples

| **Sample** | **PEG Concentration (ng/mL)** |
| --- | --- |
| PEG | 1343.9 ± 1077.9 |
| PEG 10x dil. | 816.8 ± 386.2 |
| PEG 100x dil. | 0.6 ± 0.1 |
| lPG | 0.8 ± 0.6 |
| MeOlPG | 0.5 ± 0.5 |
| lPG-block-MeOlPG | 0.7 ± 0.4 |
| MeOlPG-block-lPG | 0.7 ± 0.5 |

**Table S4:** Cell Viability of HepG2 cells when incubated with PG- and PEG-LNPs at varying mRNA concentrations as determined via CC8-assay

| **Sample** | **mRNA Concentration (µg/mL)** | | | |
| --- | --- | --- | --- | --- |
|  | **1.00** | **0.50** | **0.10** | **0.05** |
| PEG | 95.6 ± 4.9 | 89.3 ± 2.2 | 99.2 ± 4.1 | 94.8 ± 8.7 |
| lPG | 90.4 ± 18.4 | 93.8 ± 2.5 | 85.0 ± 18.8 | 87.7 ± 17.0 |
| MeOlPG | 92.4 ± 11.1 | 98.6 ± 5.3 | 91.6 ± 8.4 | 90.6 ± 21.0 |
| lPG-*b*-MeOlPG | 95.4 ± 8.6 | 94.1 ± 2.6 | 92.8 ± 8.6 | 92.1 ± 12.1 |
| MeOlPG-*b*-lPG | 95.4 ± 19.7 | 95.4 ± 4.4 | 95.8 ± 8.6 | 91.4 ± 10.2 |
| 2% SDS | 0.5 ± 0.6 | | | |

**Table S5:** Mean Fluorescence Intensity of eGFP as measured after 24 h transfection of eGFP mRNA encapsulated by PG- or PEG-LNPs in HepG2 cells on a plate reader (MFI per well)

| **Sample** | **1.00 µg/mL** | **0.50 µg/mL** | **0.25 µg/mL** | **0.125 µg/mL** | **0.0625 µg/mL** |
| --- | --- | --- | --- | --- | --- |
| PEG | 28080.9 ± 10840.9 | 27926.6 ± 10115.3 | 29078.6 ± 8476.6 | 27815.6 ± 9217.5 | 29244.9 ± 8497.6 |
| lPG | 26435.2 ± 9652.3 | 27230.6 ± 8271.4 | 25261.9 ± 7278.5 | 26321.9 ± 9895.4 | 25895.2 ± 8350.2 |
| MeOlPG | 22693.2 ± 6016.6 | 27900.6 ± 8713.8 | 27618.9 ± 8198.5 | 28536.2 ± 8162.5 | 26543.2 ± 7466.5 |
| lPG-*block*-MeOlPG | 27017.2 ± 8375.3 | 28122.9 ± 8931.5 | 28354.6 ± 8496.1 | 29320.9 ± 7746.2 | 28360.6 ± 6893.6 |
| MeOlPG-*block*-lPG | 25070.6 ± 11398.9 | 25457.9 ± 10238.0 | 25967.6 ± 9698.5 | 26765.2 ± 8694.2 | 25612.9 ± 8021.3 |

**Table S6:** Median Fluorescence Intensity of eGFP as measured after 24 h transfection of eGFP mRNA encapsulated by PG- or PEG-LNPs in HepG2 cells on a flow cytometer (MFI per cell)

| **Sample** | **1.00 µg/mL** | **0.50 µg/mL** | **0.10 µg/mL** | **0.05 µg/mL** |
| --- | --- | --- | --- | --- |
| PEG | 3481.8 ± 467.3 | 3354.5 ± 214.7 | 3637.7 ± 597.4 | 3305.3 ± 53.1 |
| lPG | 3068.0 ± 56.0 | 3890.0 ± 758.9 | 3487.3 ± 233.3 | 3695.2 ± 665.8 |
| MeOlPG | 3177.0 ± 204.9 | 3878.0 ± 660.8 | 3090.3 ± 335.1 | 3659.8 ± 539.8 |
| lPG-*b*-MeOlPG | 3424.0 ± 128.4 | 3195.2 ± 548.1 | 3485.3 ± 1086.1 | 3603.0 ± 435.9 |
| MeOlPG-*b*-lPG | 2945.0 ± 7.1 | 3816.0 ± 718.4 | 3254.0 ± 375.6 | 3590.2 ± 625.9 |

**Table S7:** Percentage of eGFP expressing cells as measured after 24 h transfection of eGFP mRNA encapsulated by PG- and PEG-LNPs in HepG2 cells on a flow cytometer

| **Sample** | **1.00 µg/mL** | **0.50 µg/mL** | **0.10 µg/mL** | **0.05 µg/mL** |
| --- | --- | --- | --- | --- |
| PEG | 38.1 ± 3.7 | 40.2 ± 3.0 | 29.7 ± 6.7 | 42.6 ± 1.3 |
| lPG | 36.2 ± 0.6 | 31.4 ± 6.4 | 43.5 ± 1.7 | 30.0 ± 7.9 |
| MeOlPG | 42.8 ± 2.5 | 37.7 ± 8.8 | 37.1 ± 8.4 | 32.5 ± 10.6 |
| lPG-block-MeOlPG | 32.9 ± 6.3 | 30.6 ± 8.4 | 21.1 ± 4.1 | 39.4 ± 12.6 |
| MeOlPG-block-lPG | 37.5 ± 2.8 | 32.2 ± 10.1 | 26.1 ± 15.4 | 27.5 ± 9.2 |
